# A review of fundamentals, challenges, prospects, and emerging trends in hydrate-based desalination

**DOI:** 10.1038/s41545-025-00484-0

**Published:** 2025-06-12

**Authors:** Ali Jalili, Georgios Kolliopoulos

**Affiliations:** Department of Mining, Metallurgical, and Materials Engineering, Université Laval, Québec, QC Canada

**Keywords:** Chemical engineering, Nanoscience and technology, Water resources

## Abstract

Hydrate-based desalination (HBD) has emerged as a promising technology among conventional desalination methods due to its low energy consumption, wide operating window with regards to total dissolved solids (TDS), and efficient water recovery. This paper provides an in-depth review of the fundamental properties of hydrates, including thermodynamic and kinetic aspects of their formation. Then, it delves into recent advancements in thermodynamic and kinetic hydrate promoters that aim to address HBD’s main challenge, which is the slow hydrate formation process. Subsequently, the review systematically examines environmental and toxicity concerns associated with chemicals used in HBD, addressing the growing demand for sustainable and biodegradable desalination solutions. Finally, a comparative analysis between HBD and conventional methods highlights its potential as an energy-efficient and selective desalination process poised to enhance sustainability within the water-energy-environment nexus.

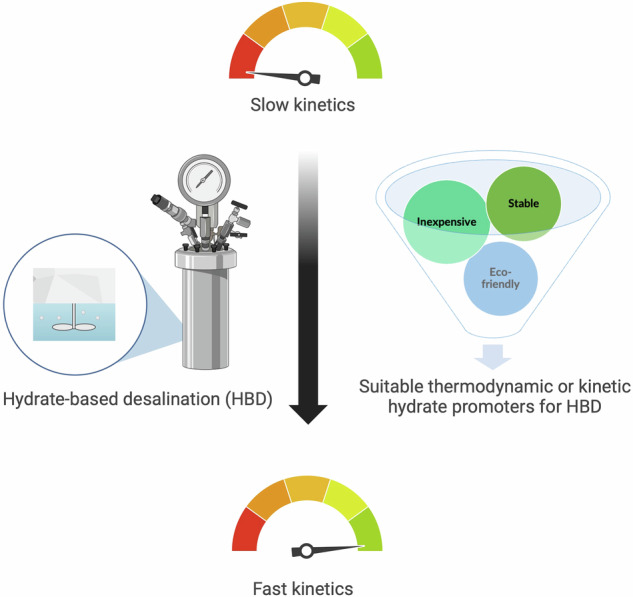

## Introduction: The concept of hydrate-based desalination (HBD)

**Fig. 1:**
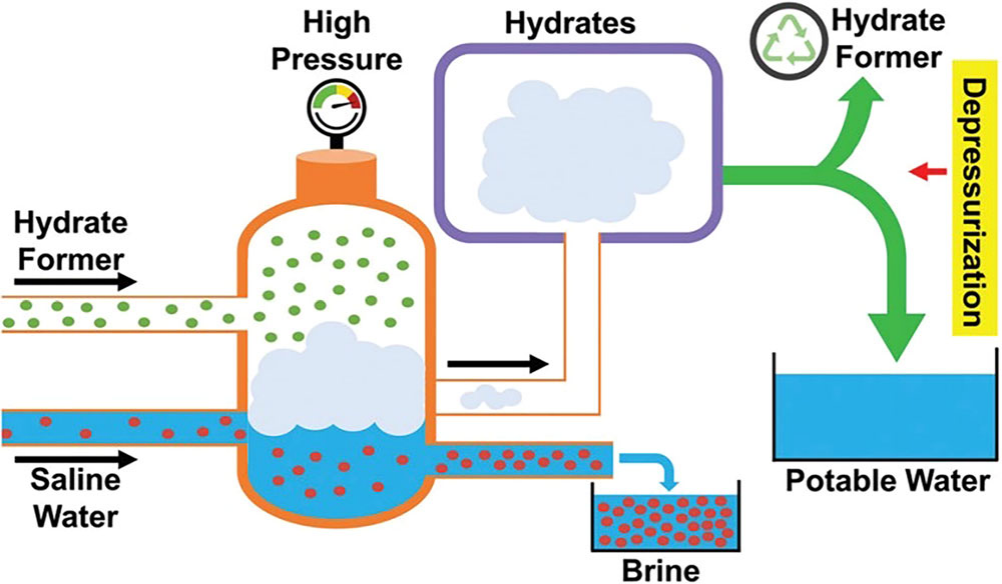
Conceptual HBD process flow diagram. Reproduced with permission from the Publisher^455^.

Water is essential for drinking, agriculture, and industry, making it a critical resource for sustaining life and prosperous development. However, global water scarcity is becoming increasingly severe, with many regions facing droughts due to climate change and population growth, which exacerbate water shortages. Sustainable desalination offers a promising solution to address water scarcity by efficiently converting seawater into clean water. Hydrate-based desalination (HBD) has been proposed as a promising process for water and wastewater treatment involving a phase change of water from liquid to solid. In HBD, a saline feed solution is exposed to gas hydrate formation conditions, which in the presence of a gas hydrate former leads to the formation of solid gas hydrate crystals. Most salts and ions are excluded from the formed hydrate crystals, which typically consist of 85–94% water and 6–15% gas hydrate former^1^; dissolved salts and ions remain in the brine solution left behind^2–5^. The hydrates formed can then be separated from the brine using mechanical methods, such as centrifugation and extrusion, and be decomposed to produce clean water via depressurization, thermal stimulation, or a combination of the above. The hydrate former can also be recovered and recycled in successive cycles of HBD. A conceptual HBD process flow diagram is presented in Fig. 1.

HBD, first proposed in the 1940s, has emerged as a promising technology to address global water scarcity, on which research intensified since 1960s and 1970s^6,7^. This technology demonstrates enhanced energy efficiency under mild conditions, reducing energy demand, membrane fouling constraints, and corrosion risks compared to conventional methods^8–11^. Early research focused on hydrate formers, demonstrating industrial-grade water recovery and partial salt removal in laboratory settings^12^. Criteria for selecting hydrate formers were subsequently established, emphasizing environmental acceptability, cost-effectiveness, stability, and non-toxicity^13^.

Recent advancements in laboratory research aimed at addressing key challenges of HBD, including optimizing hydrate formation conditions, enhancing formation kinetics, and reducing energy consumption. Extensive research concentrated on selecting hydrate formers to address challenges in salt removal, slow kinetics, and guest recovery^14–18^. Additionally, small quantities of specific additives have been shown to accelerate hydrate formation rates and/or modify thermodynamic parameters, facilitating hydrate formation at higher temperatures and/or lower pressures^19–22^. Furthermore, recent advances in micro-nano bubble (MNB) technology offer a dual solution to environmental and kinetic challenges. Unlike conventional chemical promoters, MNB technology leverages physical mechanisms, such as enhanced mass transfer and shock wave-induced radical generation, to accelerate hydrate formation while avoiding the generation of harmful byproducts or contaminants in the system^23–27^. This approach aligns with sustainability goals by minimizing secondary pollution and enabling integration with renewable energy sources^24,25^. Meanwhile, the integration of liquefied natural gas (LNG) cold energy has emerged as a promising strategy to achieve lower operating temperatures, mitigate refrigeration costs, and optimize specific energy consumption in the HBD process^28,29^. Recent modeling studies on HBD processes employing propane as the hydrate-forming agent by He et al.^1^ and Chong et al.^30^ suggest that integrating LNG cold energy with HBD is a promising large-scale alternative to current leading desalination technologies. These recent advances have achieved notable outcomes such as desalination at costs 50% lower than conventional technologies^7^, with a clean water cost of 0.148 $\frac{{\rm{USD}}}{{{\rm{m}}}^{3}}$, significantly lower than that of conventional methods^31^, water recovery rates between 30% and 70%, salt removal efficiencies of up to 98.4%^32^, treatment of hypersaline and industrial effluents, and even opened new applications beyond desalination such as liquid mining for resource recovery^33,34^. Additionally, recent innovative designs have positioned HBD as a transformative alternative to conventional desalination technologies, with the potential to fundamentally redefine established processes^35^.

Knox et al.^36^ pioneered the development of the first desalination plant using propane as a hydrate former, followed by pilot facilities advanced by Koppers Co. and Sweet Water Development Co. in the 1960s, with support from the United States Office of Saline Water^6,37^. Between 1960 and 1970, several pilot-scale processes were introduced to address challenges such as hydrate crystal separation from brine and removal of dissolved hydrate former gas from recovered water^38,39^. Later, in the 1990s, the Bureau of Reclamation worked with Thermal Energy Systems Inc., to construct pilot plants in Hawaii and San Diego using R141b as a hydrate former^40–43^. Despite these historical efforts, challenges like small dendritic hydrates, inefficiencies in separation, and low desalinated water yields persisted, alongside gaps in design parameter knowledge and equilibration principles^7,35,43^. Although advancements in filterability, reactor design, and alternative hydrate formers have addressed some issues, technical and economic barriers have limited HBD to laboratory demonstrations or prototype stages. Full-scale commercialization still faces challenges, such as unfavorable formation conditions, energy-intensive refrigeration, slow kinetics, salt entrapment, and difficulties in crystal separation from the concentrated brine^1,34,44–49^. Despite the aforementioned challenges, recent breakthroughs have ignited renewed optimism for overcoming these challenges and achieving HBD’s ultimate goal, which is large-scale commercialization.

### Salt rejection

Salt rejection or ion removal from the feed water is the objective of any desalination process. The efficiency of this rejection, associated with each salt or ion, can be estimated by Eq. (1)^7,29,50,51^:

1 $${\rm{Salt}}\,{\rm{rejection}}\, \% =\frac{{\rm{Concentration}}\,{\rm{of}}\,{\rm{the}}\,{\rm{salt}}\,{\rm{in}}\,{\rm{the}}\,{\rm{feed}}\,{\rm{water}}-{\rm{Concentration}}\,{\rm{of}}\,{\rm{the}}\,{\rm{salt}}\,{\rm{in}}\,{\rm{the}}\,{\rm{produced}}\,{\rm{water}}}{{\rm{Concentration}}\,{\rm{of}}\,{\rm{the}}\,{\rm{salt}}\,{\rm{in}}\,{\rm{the}}\,{\rm{feed}}\,{\rm{water}}}\times 100$$

Salt rejection is influenced by several parameters, including the properties of the hydrate formers^52^, operating temperature and pressure^53–56^, salinity^57,58^, ionic charge and size^52,53,57,59–62^, and additional treatment steps such as washing, centrifugation, sweating, and pelletizing^63–65^. An important consideration is that while washing can increase the salt removal, it requires additional clean water, which decreases the yield of water recovery. Ling et al.^33^ addressed this issue by using a multi-step desalination process, demonstrating that salt rejection could be increased from 82.91% in a single-step treatment to 97.41% in a two-step process, and exceed 99.00% when using three or more steps.

**Fig. 2:**
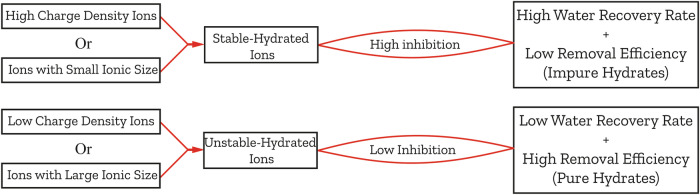
Correlation between ion removal efficiency and water recovery in HBD.

Water recovery is inversely proportional to removal efficiency, indicating that there is a delicate balance between them that determines the overall performance of HBD in terms of yield and purity of the desalinated water^32^. For instance, ions with high charge density, which promote stronger dipole-ion interactions, or those with smaller ionic sizes facilitating ion entrapment within smaller cages, contribute to the formation of stable hydrated ions. These ions, with robustly attached shell hydrates, can be eliminated during the HBD process, thereby increasing water recovery. However, the presence of such ions within the hydrates increases the concentration of impurities in the resulting clean water, diminishing the salt rejection, as illustrated in Fig. 2. This further underscores the intricate balance between water recovery and impurity removal in HBD.

### Water recovery

Water recovery is defined as the volumetric ratio of the water converted into gas hydrates to the initial water present in the feed solution and is typically less than 1. Water recovery relies on the kinetics of hydrate formation associated with the feed water concentration, hydrate former, stirring mechanism, and the separation efficiency of the hydrates from the resulting brine (F_h_)^51^. For a typical one stage HBD process, water recovery is calculated using Eq. (2)^51^:

2 $${Water\,recovery}=\frac{({Volume\,of\,water\,converted\,to\,hydrate})\times {F}_{h}}{{Volume\,of\,feed\,solution}}$$

In practice, an ideal water recovery of 1 is often not achieved due to various factors, such as the kinetics of hydrate formation and separation inefficiencies. As such, the maximum water recoverable is limited by the eutectic composition of the feed water for any desalination technology^51^.

This paper offers an updated and holistic perspective on HBD, addressing fundamental principles, progress at laboratory and pilot scales, thermodynamic and kinetic enhancement strategies (e.g., novel promoters such as nanobubbles and nanoparticles, and LNG cold energy integration), techno-economic analyses, toxicity impacts of the chemical components and environmental concerns of the process, current challenges, and future prospects. Additionally, a comprehensive review of recent investigations on the application of hydrate-based technologies in the desalination of various feed waters, highlighting their potential through the use of different enhancing methods, such as THPs and KHPs, as well as other innovative substances or apparatus is presented in this review paper. By synthesizing recent advancements and presenting unresolved barriers, this review aims to guide future research and development efforts in the field of HBD.

## Fundamental insights into hydrates and recent innovations in HBD

### Hydrates and their structures

Hydrates are fascinating structures characterized by non-stoichiometric crystalline arrangements where some cavities remain vacant while others are occupied^42,66–68^. These formations, known as clathrate compounds (derived from the Latin term “clathratus,” meaning “encaged”), typically manifest at elevated pressures and low temperatures (generally above the freezing point of water), and in the presence of sufficient water molecules and hydrate formers^51,69,70^. In these structures, small guest molecules known as hydrate formers, typically less than 10 Å in size^71^, are trapped inside cages formed by hydrogen-bonded water molecules, which range from 0.395 to 0.586 nm^2,42,66,72^. It is important to note that a perfect hydrate crystal, where all the cavities within its cages are fully occupied, does not exist^73^. Guest molecules are not chemically bonded to water molecules; instead, they interact through weak van der Waals forces^49,74^ forming a crystalline solid compound that is physically similar to ice under certain conditions^37,75^.

**Fig. 3:**
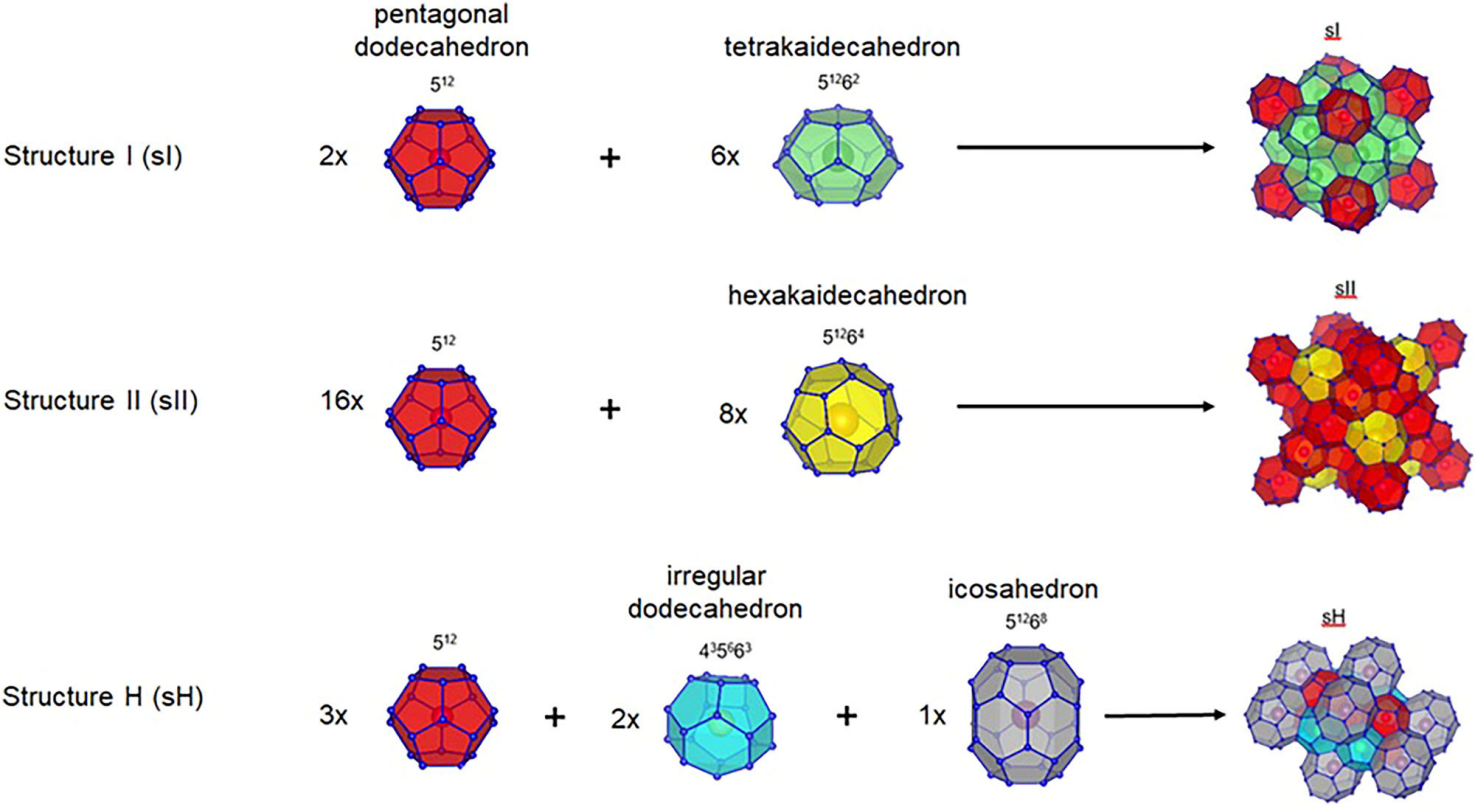
A visual representation of gas hydrate structures. Reproduced with permission from the Publisher^456^.

The shape, type, and size of guest molecules significantly influence the structure of hydrates, resulting in cages of varying sizes and shapes^73,76–78^. Hence, three primary cage configurations, known as cubic structure I (sI), cubic structure II (sII), or hexagonal structure H (sH), are notable for gas hydrates^37,51,75,79^. These structures consist of convex polyhedrons interconnected through vertices or face-sharing in three dimensions or via face-sharing in two dimensions, which form the hydrate structures^80,81^. The cavities within these structures vary in size and typically accommodate one guest molecule per cavity, excluding hydrogen molecules^81^. Guest molecules have the ability to rotate within the cages, inducing distortions, although they cannot diffuse between cages^81^. Not all cavities need to be occupied by guest molecules; occupancy is influenced by the size of the guest molecules, as well as by pressure, temperature, and system composition^81^. To aid in understanding these structures, various methods have been proposed, with Jeffrey’s representation being particularly useful^82^. This technique employs the notation ‘m^n^’ to denote cavities, where “m” signifies the number of edges on a specific polyhedron face, and “n” indicates the quantity of that specific face within the polyhedron cavity^82^. For instance, a pentagonal dodecahedron comprises of 12 (n = 12) pentagonal (m = 5) faces which can be illustrated like (5^12^). Figure 3 presents a comprehensive schematic representation of hydrate structures, while Table 1 provides a concise summary of their key characteristics, including examples of their formers in a practical chart.

**Table 1:** Principal characteristics of hydrate structures

Features							
Hydrate Structure	Crystal structure	Number of cavities/ideal unit cell	Number of water molecules/ideal unit cell	Average cavity radius (Å)	Guest molecule		References
sI	Cubic	Small: 2 (5^12^) Large:6 (5^12^6^2^)	46	Small: 3.95 Large: 4.33	H_2_S, CO_2_, C_2_H_6_, CH_4_		^37,51,66,79,354–356^
sII	Cubic	Small: 16 (5^12^) Large: 8 (5^12^6^4^)	136	Small: 3.91 Large: 4.73	C_3_H_8_, HC(CH_3_)_3_, Ar, Kr, O_2_, N_2_, SF_6_		^1,49,66,79–81,357–362^
sH	Hexagonal	Small: 3 (5^12^) Medium:2 (4^3^5^6^6^3^) Large: 1 (5^12^6^8^)	34	Small: 3.94 Medium: 4.04 Large: 5.79	**Gas helper**	**Primary gas**	^37,66,78,79,363^
					CH_4_		
					Xe	C_5_H_10_	
					H_2_S	C_6_H_12_	
					N		

### Thermodynamic analysis of hydrates

**Fig. 4:**
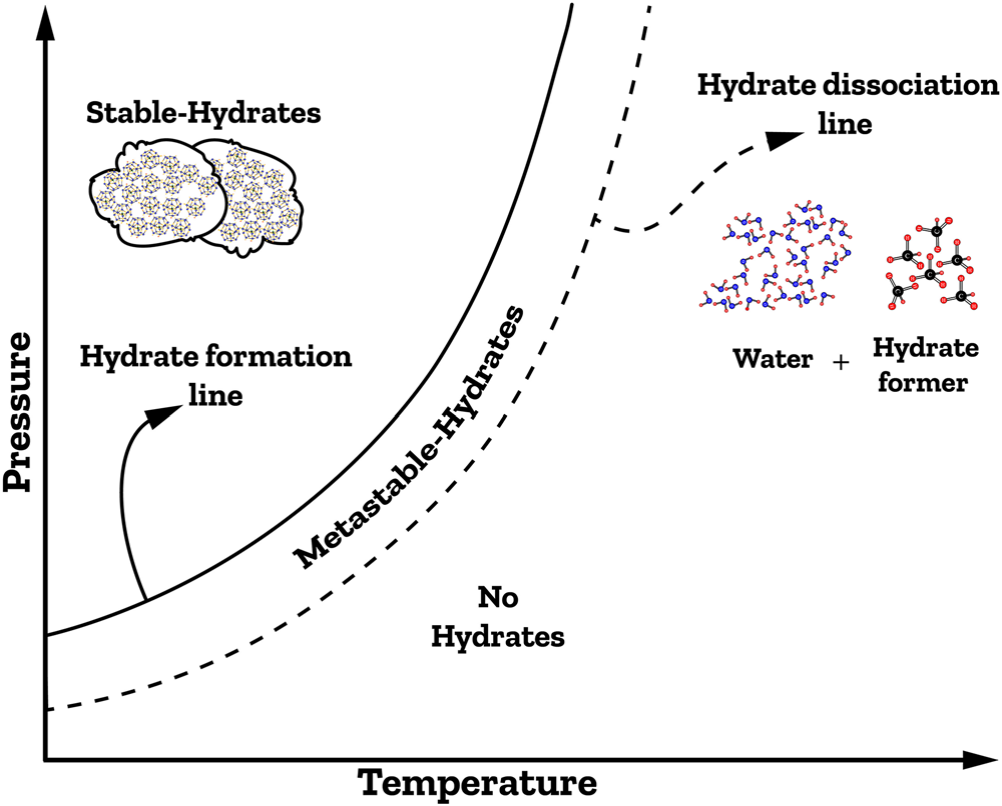
Hydrate equilibrium curves.

Predicting the conditions for gas hydrate formation or dissociation using thermodynamic models can significantly advance the development and implementation of hydrate-based technologies in the industry. Generally, thermodynamic models for predicting hydrate phase equilibria fall into two categories: van der Waals and Platteuw (vdW–P)-based models and Chen–Guo-based models^83^. Historically, it was Barrer and Stuart who first determined the properties of gas hydrates through utilizing a statical thermodynamic approach in 1959^73,84^. Subsequently, Waals and Platteuw stated a statistical thermodynamic model, which was based on classical adsorption theory and the difference between the chemical potential of water in the hydrate phase (μ_Hw_) and a hypothetical empty lattice hydrate phase (μ_βw_)^73^. Numerous scientists have built upon this model to develop their own. For instance, Saito et al.^85^ established a model to predict gas hydrate equilibria by equating the chemical potential of water in the hydrate and aqueous (or ice) phases. This model was later generalized by Parrish and Prausnitz^86^. Another notable thermodynamic model for predicting hydrates is the Chen–Guo model^87^. By 1998, advancements in modeling allowed the inclusion of complex systems with electrolytes and alcohols like glycerol and methanol^32,88^. This progress facilitated the creation of models predicting gas hydrate formation under conditions involving electrolyte mixtures, including NaCl, KCl, and CaCl_2_^32,89^. Fig. 4 shows a hydrate equilibrium phase diagram, consisting of regions associated with gas hydrate formation and dissociation, based on experimental observations and theoretical calculations. The composition of hydrate formers and additives can shift the hydrate equilibrium curves and functional operating conditions. In Fig. 4, the regions to the right of the dashed line display conditions where both water molecules and hydrate formers can coexist. If solid hydrates are subjected to the conditions in these regions, they will dissociate and breakdown into their constituent water and hydrate-forming molecules. Consequently, the dashed line can be referred to as the dissociation line, and the zones on the right side of this line as dissociation areas. Conversely, to the left of this dissociation line, hydrates are thermodynamically stable and have the potential to form. Their formation depends on the presence of a driving force for hydrate formation, which can be explained by the existence of a metastable zone between the solid line and the dashed line; hydrates can indeed exist in this region. It is important to note that salts, which are significant components in various types of feed water, act as thermodynamic inhibitors in hydrate-based procedures. The presence of salts shifts the hydrate equilibrium and influences hydrate formation conditions, necessitating higher pressures and lower temperatures due to the Coulombic effect^37,52^.

### Hydrate formation and its kinetics

**Fig. 5:**
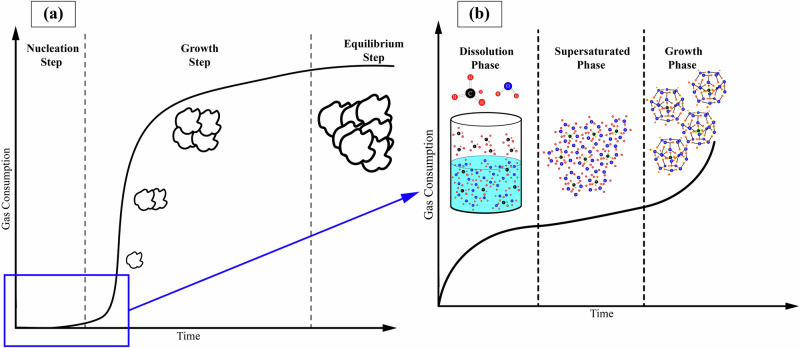
Typical time-dependent behavior of the hydrate crystallization procedure. **a** Schematic representation of the kinetic progression of gas consumption over time during the formation of gas hydrates can be divided into three distinct stages: Stage I, known as the nucleation stage; Stage II, referred to as the growth stage; and Stage III, known as equilibrium. Reproduced with permission from the Publisher^32^. **b** Magnified section of Stage I and visual representation of the different processes taking place during the nucleation stage. Reproduced (adapted) with permission from the Publisher^35^.

Gaining a profound understanding of hydrate formation kinetics is crucial for determining the hydrate formation rate and improving the efficiency of hydrate formation^35^. Hydrate formation can be monitored by assessing the consumption rate of the hydrate formers and their transformation into crystalline gas hydrate structures^90^. Fig. 5a illustrates a schematic of gas consumption versus time for an agitated system at constant temperature and pressure, which involves the continuous addition of gas hydrate formers^32^. The hydrate formation process can be divided into three stages: nucleation, growth, and equilibrium. Hydrate nucleation can occur either homogeneously, without impurities, or heterogeneously, in the presence of impurities or foreign surfaces. Homogeneous nucleation is rare in nature and involves only the gas hydrate former and liquid, while heterogeneous nucleation is facilitated by impurities. As illustrated in Fig. 5b, initially, gas dissolves into the liquid, and once the solution becomes supersaturated, favorable temperature and pressure conditions lead to the clustering of water molecules around dissolved gas molecules, forming complete or incomplete crystal embryos. These embryos must then grow beyond a critical size during the induction time to stabilize; otherwise, smaller embryos dissolve back into the solution. Embryos continuously form, grow, and shrink due to local changes in mass, pressure, and temperature, making nucleation a free energy-dependent stochastic process. The induction time is the period from the onset of hydrate formation to the initial nucleation, marked by a swift increase in temperature (due to the exothermic nature of hydrate formation) and a decrease in pressure due to the formation of gas hydrates^35,91,92^. This duration, varying from a few minutes to multiple days, depends on factors such as the gas type, temperature, pressure, concentration, and the apparatus used^32,73,75^. Once embryos surpass the critical radius, they act as potential nuclei for the growth stage, the second stage of the hydrate formation process, which is marked by an increase in gas intake^35,93,94^. Hydrate growth is characterized by the progressive increase in both size and number of self-sustaining hydrate nuclei. Due to the molecular scale of both nucleation and initial growth, delineating a clear boundary between them is challenging^95^. During the growth stage, significant hydrate formation is macroscopically observable as hydrates expand rapidly until equilibrium is attained (i.e., the third stage of the hydrate formation process)^32,95^. A critical factor in hydrate formation is the heat released during this exothermic process, which, along with gas consumption, can reduce pressure, potentially pushing the system towards metastability. Conversely, the heat required for the endothermic desalination of hydrates, along with the production of clean water and the release of free gas, can also drive the system back towards metastability. Therefore, maintaining a delicate balance of conditions is essential for both the formation and dissociation processes^96^. A comprehensive review of nucleation theories and growth models, including an in-depth discussion on the three major controlling mechanisms (intrinsic kinetics, mass transfer limited, heat transfer limited) for hydrate growth has been presented elsewhere^37,95^.

With the consumption of available water, the rate of gas consumption decreases. Indeed, the rate of gas consumption is closely tied to the quantity of water available. Initially, when ample water is present to form hydrates with the gas hydrate former, the gas consumption rate is high. However, the gas consumption rate decreases as hydrate formation progresses and the volume of available water diminishes. To compute the rate of hydrate formation, it is essential to quantify the consumption of the gas hydrate former throughout the process. As gas is consumed at a constant temperature, the pressure within the closed system decreases. The total moles of gas, encompassing gas moles in the hydrates (${n}_{H}$), dissolved in water (${n}_{w}$), and in the gas phase (${n}_{G}$), remains constant and equals the initial amount at the beginning of the process^94^. Consequently, the amount of consumed gas at any given time, t, can be determined by the difference between the number of gas moles in the gas phase at the start (${{\rm{n}}}_{{\rm{H}},0}$) and at the selected time, t, (${{\rm{n}}}_{{\rm{H}},{\rm{t}}}$)^94^. The moles of the consumed gas ($\Delta {{\rm{n}}}_{{\rm{H}},\downarrow }$) can be measured by Eq. (3)^94^:

3 $$\triangle {{\rm{n}}}_{{\rm{H}},\downarrow }={{\rm{n}}}_{{\rm{H}},{\rm{t}}}-{{\rm{n}}}_{{\rm{H}},0}={\left(\frac{{\rm{PV}}}{{\rm{zRT}}}\right)}_{{\rm{G}},0}-{\left(\frac{{\rm{PV}}}{{\rm{zRT}}}\right)}_{{\rm{G}},{\rm{t}}}$$

where G represent the guest molecule phase (gas), $\triangle {{\rm{n}}}_{{\rm{H}},\downarrow }$ is the moles of the consumed gas, Z is the compressibility factor calculated by Pitzer’s correlation^97^, R, T, V and, P are the gas constant, temperature, gas phase volume, and pressure, respectively. The negative amount of $\triangle {{\rm{n}}}_{{\rm{H}},\downarrow }$ indicates that gas is being consumed during the hydrate formation process. To account for variations in sample size, the total number of gas molecules and the amount of gas consumed are typically normalized. This normalization represents the total volume of gas trapped in one mole of the water in the system, as shown in Eq. (4)^25,73^:

4 $${n}_{N}=\frac{\triangle {{\rm{n}}}_{{\rm{H}},\downarrow }}{{n}_{w}}$$

Therefore, the rate of hydrate formation can be calculated by utilizing the forward difference method, as presented in Eq. (5)^94^:

5 $${\left(\frac{{\rm{d}}\triangle {{\rm{n}}}_{{\rm{H}},\downarrow }}{{\rm{dt}}}\right)}_{{\rm{t}}}=\frac{{(\triangle {{\rm{n}}}_{{\rm{H}},\downarrow })}_{{\rm{t}}+\triangle {\rm{t}}}-{(\triangle {{\rm{n}}}_{{\rm{H}},\downarrow })}_{{\rm{t}}}}{\triangle {\rm{t}}}$$

Additionally, the water-to-hydrate conversion ratio (${{\rm{C}}}_{{\rm{W}}\to {\rm{H}}}$) is another critical determinant in the kinetics of hydrate formation that quantifies the fraction of water molecules transformed into gas hydrate per mole of the initial solvent^35,73^. Eq. (6) is used to determine ${{\rm{C}}}_{{\rm{W}}\to {\rm{H}}}$^34^$.$

6 $${C}_{W\to H}=\frac{{\triangle {{\rm{n}}}_{{\rm{H}},\downarrow }\times h}^{n}}{{n}_{{H}_{2}0}}$$

where $\triangle {{\rm{n}}}_{{\rm{H}},\downarrow }$ represents the gas (i.e., hydrate former) consumed to form hydrates, which can be computed using Eq. (3). The term ${{\rm{n}}}_{{{\rm{H}}}_{2}0}$ corresponds to the moles of water present in the reactor. Furthermore, ${{\rm{h}}}^{{\rm{n}}}$ denotes the hydration number, which is defined as the quantity of water molecules required to clathrate a single molecule of the hydrate former^35,51,73^.

## Insights into thermodynamic hydrate promoters and kinetic hydrate promoters in HBD

A primary challenge in HBD is the sluggish kinetics associated with gas hydrate formation^32^. During the growth phase, the kinetics of gas hydrate formation are predominantly governed by heat and mass transfer^98,99^. In contrast, nucleation, which is the initial stage of hydrate formation, is primarily influenced by factors such as supersaturation, interfacial energy, subcooling, pressure, and the presence of impurities or promoters, rather than heat and mass transfer. This section will explore various strategies and methods to mitigate the slow kinetics inherent to gas hydrate formation, encompassing both nucleation and growth phases.

Chemical promoters are used to milden the conditions or expedite hydrate formation, thus aiding in overcoming some of the inherent challenges associated with the slow kinetics of hydrate-based technologies. Although the presence of promoters is not essential for hydrate formation, they can significantly assist the process. Slow kinetics and high operating costs of hydrate-based technologies are challenges that researchers aim to address using these chemical substances. Selecting appropriate and practical promoters is a critical initial step in achieving favorable hydrate formation kinetics and thermodynamic behaviors for HBD and optimizing the efficiency and cost-effectiveness of the process. Suitable candidates should accelerate the nucleation and growth kinetics of gas hydrates to reduce processing times. Furthermore, compatibility with water, stability, minimal toxicity, biodegradability, and reusability are essential properties of promoters in order to minimize both operating costs and the process’ environmental impact.

Overall, chemical promoters can be categorized into two major categories: thermodynamic hydrate promoters (THP) and kinetic hydrate promoters (KHP). THPs and KHPs can often be mixed to gain the advantages of each category, while also reducing each one’s flaws^100–107^. The following sections will delve into each group of promoters, namely THPs and KHPs, and will analyze their recent advancements.

### State-of-the-art and recent advanced THPs

**Fig. 6:**
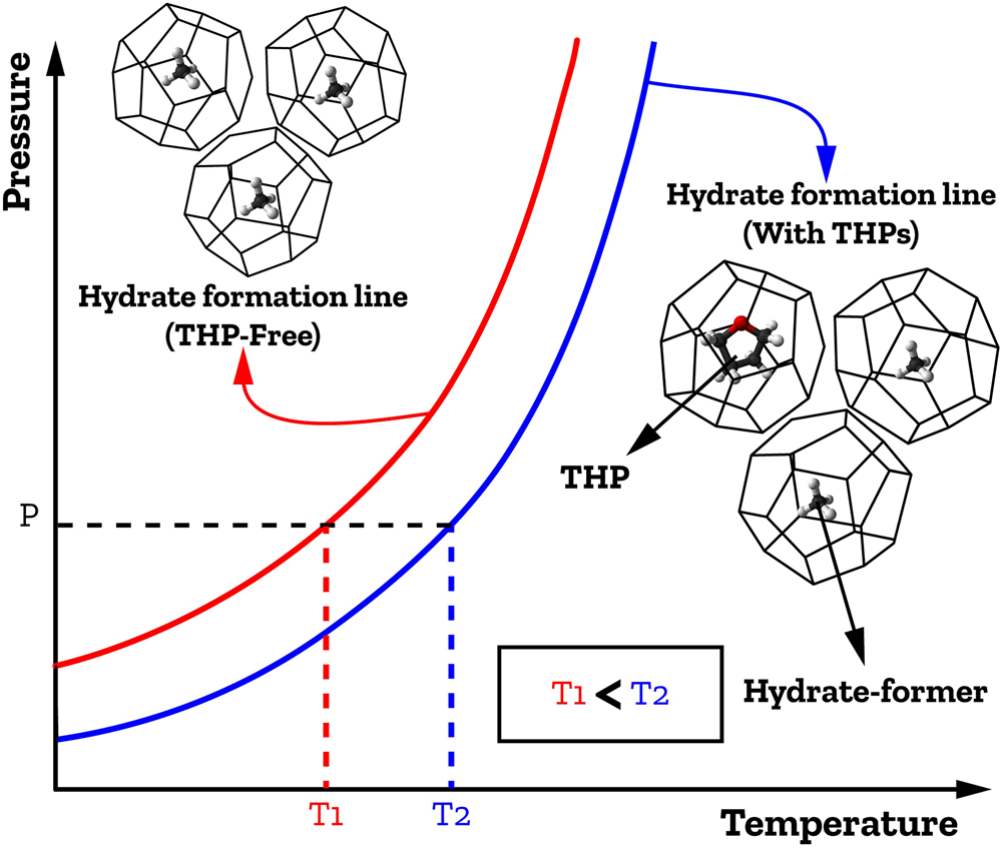
Schematic effects of THPs on gas hydrate equilibrium curves.

THPs are a significant category of chemical additives that can be used to modify the conditions required to form hydrates, and shift the hydrate equilibrium curve to milder conditions, a rightward shift to lower pressures and higher temperatures, as shown in Fig. 6, thereby improving the overall efficiency of hydrate formation. This increased driving force for gas hydrate formation facilitates hydrate nucleation thus accelerating hydrate formation^108–111^.

THPs can be categorized into two distinct types: thermodynamic clathrate hydrate promoters and thermodynamic semi-clathrate hydrate promoters. The first group consists of small molecules, i.e., tetrahydrofuran (THF), propane (C_3_H_8_), acetone ((CH_3_)_2_CO), and cyclopentane (CP), which occupy water cavities within the hydrate structures, leading to the formation of stabilized hydrates and improved growth rates^22,73,112^:

#### Tetrahydrofuran (THF, C_4_H_8_O)

Due to its larger molecular size and capability to occupy large cages within the hydrate structure, THF effectively stabilizes hydrates while shifting equilibrium to milder conditions^113^. THF is able to significantly reduce hydrate phase equilibrium formation pressures at any temperature, with the most substantial reduction observed at a stoichiometric concentration of 5.56 mol%^73,114^. Majid et al.^22^ reported shifts in hydrate equilibrium curves of 4–20% for CH_4_ and 2–20% for CO_2_ at constant pressure, with larger shifts noted at lower pressures. Lee et al.^115^ studied the CO_2_ + H_2_ mixture using varying concentrations of THF and found that ~1 mol% THF maximized the gas uptake while reducing induction times; however, higher concentrations led to a reduction in the formation rates due to concentration dependencies. This optimum is system-specific and depends upon the hydrate former as well as the subcooling of the system. In another study, Sabil et al.^116^ investigated the THF’s impact on hydrate formation kinetics using a laboratory-scale reactor by comparing single CO_2_ hydrates with mixed CO_2_ + THF hydrates. Their findings showed that including THF reduced induction times, increased apparent rate constants for formation, enhanced nucleation processes, and accelerated growth onset compared to using CO_2_ alone.

#### Propane (C_3_H_8_)

This is a nontoxic hydrocarbon that can also serve as an effective promoter of hydrate formation, leading to significant enhancements in both the stability and hydrate formation conditions^117,118^. He et al.^119^ reported that, CO_2_ combined with C_3_H_8_ exhibited superior performance, resulting in lower formation pressures and faster kinetics, achieving higher water-to-hydrate conversion in shorter timeframes. Kumar et al.^120^ investigated fuel gas mixtures (40% CO_2_ and 60% H_2_), finding that the addition of 3.2% C_3_H_8_ reduced the equilibrium formation pressure from 10.74 MPa to 5.1 MPa at 277.8 K, effectively lowering the pressure by ~50%. This study also noted a structural transition from sI to sII hydrates upon C_3_H_8_ addition. Babu et al.^121^ further confirmed these findings, demonstrating that adding 2.5% C_3_H_8_ to these mixtures at 278.4 K reduced the equilibrium pressure by 67%, from 10.74 MPa to 3.5 MPa. Their results showed that in sII hydrates, C_3_H_8_ occupied 43% of large cages, while H_2_ filled the small cavities, with CO_2_ occupying the remaining large cages. Majid et al.^22^ reviewed hydrate equilibrium curves for binary gas mixtures of CO_2_ with C_3_H_8_ concentrations ranging from 6% to 20% at pressures between 0.3 and 4.0 MPa. They observed shifts of 1–10% in equilibrium temperatures compared to pure CO₂ hydrates. In fact, incorporating small amounts of C_3_H_8_ (in most cases 2.5–3.2 mol%) into hydrate-forming gas mixtures significantly enhances thermodynamic stability and alters the structural properties of the gas hydrates^73,78,122^. Du et al.^122^ proposed a micro-formation mechanism, indicating that C_3_H_8_ exhibits a greater binding energy in sII-5^12^6^4^ cages, supported by significantly higher binding energy for C_3_H_8_ in sII-5^12^6^4^ than in sI-5^12^6^4^ cages. This implies that, although sI and sII hydrates may coexist initially, the growth rates of sI hydrates are suppressed, resulting in their gradual transformation into more stable sII hydrates as the reaction progresses. It is important to mention that while C_3_H_8_ generally promotes hydrate formation, it can also operate as a kinetic inhibitor and reduce the hydrate formation rate^123^.

#### Cyclopentane (CP, C_5_H_10_)

This is a hydrate former that can also be considered as a THP when mixed with other hydrate formers, like CO_2_^54^. This chemical substance occupies the sII-5^12^6^4^ cages and leads to the formation of hydrates under more favorable conditions. This promoting behavior can cause a rightward shift in equilibrium hydrate formation curves. Based on Cha and Seol’s work^54^, CP usage along with other hydrate formers, like CO_2_, can raise upper temperature limits by up to 16 K compared to pure CO_2_, accelerate reaction rates, improve salt rejection, enhance salt removal efficiency, and increase energy efficiency. In another study, Lv et al.^124^ demonstrated that increasing the volume ratio of CP in mixtures with CH_4_, enhances its applicability as a practical THP. Overall, when selecting promoters, it is important to recognize that achieving the necessary pressure using gas hydrate formers is generally more efficient and cost-effective than lowering the temperature. Consequently, in systems containing gas hydrate formers, THPs should ideally elevate the formation temperature (like CP) rather than reduce pressure requirements (such as C_3_H_8_)^49,54,125,126^. While gaseous hydrate formers can expedite hydrate growth under high pressure, CP exhibits slower kinetics but facilitates hydrate nucleation. Thus, combining CP with gaseous hydrate formers can help overcome the limitations associated with using single agents or liquid promoters alone, while also enhancing the yield of dissociated water^3,59^. Sun et al.^127^ examined phase equilibria involving CH_4_ and CP, illustrating a 6–27% rightward shift due to CP presence, investigations into CO_2_ hydrates with excess CP indicated smaller shifts (4–7%) within similar pressure ranges^128^. Zheng et al.’s research^129^ further confirmed the enhancing behavior of CP as a THP; however, this enhancement plateaued when the CP molar ratio exceeded 0.01. Overall, the immiscibility of CP in water can act as a double-edged sword. On one hand, upon dissociation of CP hydrates, two distinct phases emerge: pure water and liquid CP; this separation can facilitate the recovery of water while maintaining the integrity of the CP phase^59^. On the other hand, the liquid CP phase may restrict gas molecules from readily reaching the water interface, which is crucial for the nucleation and growth of hydrates, potentially slowing down hydrate formation kinetics^73^. Hence, while CP presents a significant potential as a THP in gas hydrate systems, its immiscibility with water requires careful consideration to balance its beneficial effects against potential limitations.

The typical high volatility of the compounds in the first group of THPs necessitates additional separation recovery steps to minimize losses^112^. These often costly recovery steps make the industry more inclined to use the second THP group, like non-volatile organic quaternary ammonium salts such as tetrabutylammonium bromide (TBAB), known as ionic hydrate formers. These compounds can alter conventional water cage coordination and form semi-clathrate hydrates at ambient pressure and temperature, entrapping different gases more effectively than CP and THF^22,73,112,130–133^. Semi-clathrates, a term introduced by Davidson^75^, are guest-host crystalline structures consisting of water and hydrophobic molecules. They are characterized by a partial disruption of the water cage structure, allowing ions from hydrophobic molecules to engage with the hydrate cavity and substitute water molecules at specific positions within the clathrate cages^134–137^. Unlike conventional clathrate hydrates, hydrophobic molecules that form semi-clathrates serve not only as guest molecules inside the cages but also as hosts, incorporating with water molecules into their lattice framework and leading to unique structural properties^22^. The formation of semi-clathrate hydrates is defined by their water-anion framework, which includes various large and small cavities capable of accommodating different guest molecules^35^. For example, the structure of TBAB hydrate cage is disrupted to accommodate the larger TBA⁺ cation while the Br⁻ anion participates in the hydrogen-bonded water framework^138^. Specifically, charged centers from cations and anions substitute certain positions in the hydrate lattice, while alkyl chains from salts, like TBA^+^, occupy larger cages such as 5^12^6^2^, 5^12^6^3^, or 5^12^6^4^, and smaller 5^12^ cages fill the spaces between these larger cavities^139^. This interaction, which is the key differentiator from clathrates, influences their stability and functionality in various applications^112,140,141^, such as serving as THPs^103,142–144^. However, semi-clathrates share similarities with clathrates in that they both contain hydrogen-bonded water molecule frameworks and possess cage-stabilizing guest molecules as part of their structure^103^.

#### Tetrabutylammonium bromide (TBAB, C_16_H_36_NBr)

A well-known quaternary ammonium salt is able to form semi-clathrate hydrates. In these structures, TBAB occupies large cavities (5^12^6^2^ and 5^12^6^3^), while smaller guest molecules compete for occupying relatively small cavities^75,145–147^. Notably, smaller gas molecules do not always occupy small cavities; for example, CH_4_ occupies small cavities in CH_4_-C_3_H_8_-TBAB semi-clathrate hydrates^148^, whereas in CO_2_-H_2_-TBAB systems, more CO_2_ molecules are encaged in small cavities than H_2_ molecules^112^. TBAB due to its structure and properties has been shown to shift the hydrate equilibrium curve to milder conditions, facilitating the formation of semi-clathrate structures under relatively mild hydrate formation conditions^149–152^. Li et al.^153^ illustrated that increasing TBAB concentration from 0.14 to 2.67 mol% continuously mitigated equilibrium formation pressure at certain temperatures. In another investigation of CO_2_ + TBAB mixtures, CO_2_ hydrates formed at ~0.5 MPa at 282.5 K, which corresponds to an 87% pressure reduction compared to single CO_2_ hydrates (3.86 MPa)^154^. Majid et al.,^22^ reviewed several studies on CH_4_-TBAB systems at various concentrations and pressures, concluding that there were rightward shifts of 1.5–60 K in hydrate equilibrium temperature due to TBAB usage.

Operating conditions and TBAB concentrations have a huge impact on TBAB behavior during hydrate formation. For example, it has been observed that the influence of TBAB on hydrate formation is temperature-dependent^155^: at lower temperatures, TBAB acts as a promoter, facilitating hydrate formation, whereas at higher temperatures, it exhibits inhibitory effects on the same hydrate system. Lin et al.^156^ investigated the equilibrium conditions of hydrates formed from CO_2_-TBAB-H_2_O mixtures with TBAB concentrations ranging from 4.43 to 9.01 wt%. Their analysis determined that TBAB allowed a decrease in CO_2_ hydrate formation pressure by ~74% at 283 K and 87% at 279 K, with reductions dependent on TBAB concentration. With regards to TBAB concentration, increasing TBAB concentration initially mildens the conditions for hydrate formation until stoichiometric concentrations are achieved; however, beyond this threshold, phase equilibrium conditions harshen^73,143,157^. Ma et al.^158^ observed that low concentrations of TBAB (less than 10 wt%) exhibit a more pronounced promoting effect compared to higher concentrations. This effect arises due to the formation of semi-clathrate hydrate cages, which induce the formation of neighboring CO_2_ hydrate cages, and initiate a self-adjustment process that arranges the water molecules in a more ordered manner. Conversely, at higher concentrations, the abundance of TBA^+^ at the interface generates an electric field, disrupting the formation of semi-clathrate hydrate cages. Additionally, the tightly packed arrangement of TBA^+^ at the gas-liquid interface partially inhibits the mass transfer of CO_2_, leading to lower promoting effect. Kim et al.^143^ also utilized a CO_2_-H_2_ gas mixture with various concentrations of TBAB and found that increasing TBAB concentration up to 3.0 mol% shifted phase equilibrium conditions to milder states; however, concentrations beyond this threshold resulted in increased phase equilibrium temperature and pressure, indicating a critical concentration for additive effectiveness. Mohammadi et al.^159^ further confirmed this trend, stating that exceeding a specific stoichiometric ratio of TBAB in systems led to significant inhibiting behavior.

In addition to serving as THP, TBAB is an organic salt that can enhance the kinetics of semi-clathrate hydrate formation^73,160,161^. Li et al.^162^ reviled the TBAB capability to accelerate formation of CO_2_/N_2_ hydrates with an induction time reduction from 19 min to 5 min and their formation completion within 1 h under milder conditions (277.5 K and 4.01 MPa). The hydrate formation rate constant increased with feed pressure, reaching a maximum value of 1.84 × 10^−7^ $\frac{{{mol}}^{2}}{s.J}$. In another research, Ansari et al.^163^ investigated the promoting effect of TBAB on the CO_2_ hydrate formation and found that increasing TBAB concentration above 10 wt% does not significantly impact the equilibrium condition, but at lower percentages, its effect is pronounced. Additionally, the lowest induction time was observed at the highest TBAB concentration, showing a 94.75% reduction from 5 wt% to 32 wt%. However, gas consumption increases with TBAB concentration up to 10 wt%, beyond which it decreases due to solid hydrates.

### State-of-the-art and recent advances in KHPs

**Fig. 7:**
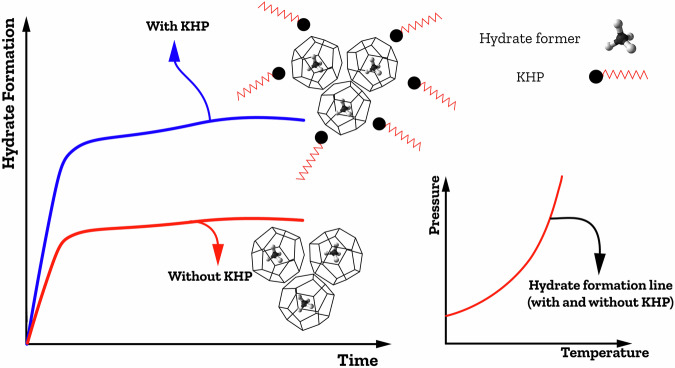
Formation of gas hydrates in the presence of a KHP. Reproduced (adapted) with permission from the Publisher^22^.

KHPs are chemical additives used at low concentrations (typically less than 10,000 ppm) to tackle the slow kinetics of hydrate formations. These promoters include surfactants as well as other high-surface materials like nanoparticles, micro- and/or nanobubbles, and amino acids^73^. They effectively lead to a decrease in induction time (thereby promoting nucleation), an acceleration of the hydrate formation process (enhancing growth rates), and an increase in gas uptake. KHPs hold promise in advancing practical application of hydrates by improving the interfacial interactions between gas and liquid phases without substantially shifting the hydrate equilibrium curve (Fig. 7). Ongoing research aims to identify and develop KHPs that can effectively enhance hydrate formation rates under economically viable conditions. This section will discuss KHPs in detail.

#### Surfactants

Surface-active agents, are compounds characterized by the presence of both hydrophobic and hydrophilic components, enabling them to dissolve both polar and non-polar substances. The distinct properties of surfactants arise from the interactions between their hydrophobic and hydrophilic segments. At appropriate concentrations, surfactant molecules aggregate in water to form structures known as micelles, which can take on different shapes and orientations, such as spherical, rod-like, or multilayered configurations. Surfactants are primarily classified into four categories based on their molecular moieties: anionic, cationic, non-ionic, and zwitterionic surfactants. Surfactants can act as either inhibitors or promoters in hydrate-based processes^164–166^. They affect surface charges and viscosity, facilitating hydrate nucleation and significantly impacting hydrate morphology by reducing the contact angle between the components, decreasing the clathrate-aqueous phase interfacial tension, and lowering the surface free energy through adsorption at the interface^167^. In systems containing surfactants, hydrate-forming gases dissolve to a higher local concentration. This, combined with the formation of molecular clusters that resemble hydrate structures^168–170^, facilitates the formation of hydrate structures. These enhancements occur through hydrophobic interactions at concentrations above the critical micellar concentration. The above results in a reduction in induction time^165^. Moreover, surfactants improve mass transfer between the nucleons and their surroundings by promoting the mixing of water and gas hydrate formers, which in turn accelerates hydrate crystal growth^165^. Karaaslan et al.^171^ conducted experiments to examine the effects of three types of surfactants, namely anionic (LABSA), cationic (DAM), and non-ionic (ETHOXALATE), indicating that these additives did not significantly alter the thermodynamic conditions for natural gas hydrate formation but did affect the hydrate formation kinetics. All concentrations of the anionic surfactant resulted in an increased overall hydrate formation rate, suggesting its potential as a KHP. Regarding the cationic surfactant, although DAM demonstrated a promoting behavior at low concentrations, less than 0.05 wt%, it exhibited the opposite effect at higher concentrations. The impact of the non-ionic surfactant on hydrate formation was less pronounced, indicating that non-ionic surfactants typically function as kinetic inhibitors rather than promoters. Therefore, there is a critical concentration that determines the influence of surfactants on hydrate formation processes. Sodium dodecyl sulfate (SDS), an anionic surfactant, has been recognized as one of the most effective surfactants in enhancing the nucleation and growth of gas hydrate crystals^165,171,172^. SDS is able to remarkably accelerate the kinetics of hydrate formation with no effect on the resulting hydrates’ structures. Liu et al.^173^ reported the existence of an optimal concentration of SDS, i.e., 0.05 wt%, for CO_2_ hydrate formation. They utilized Eq. (7) to calculate the conversion rate of water-to-hydrate^173^:

7 $$\frac{n\times \triangle {{\rm{n}}}_{{\rm{H}},\downarrow }\times {M}_{{H}_{2}O}}{m}={C}_{w}$$

where n, m, $\triangle {{\rm{n}}}_{{\rm{H}},\downarrow }$, and ${{\rm{M}}}_{{{\rm{H}}}_{2}{\rm{O}}}$ are the hydration number of hydrate former, the mass of water in the reactor (g), the gas consumption of hydrate former obtained from Eq. (3), and the molar mass of water (g/mol), respectively. Based on their report and calculations, an increase in SDS concentration from 0.01% to 0.05% led to a 94% reduction in the induction time for CO_2_ hydrate formation (from 32 min to 2 min). Additionally, the hydrate conversion rate increased by 93% (from 12.05% to 23.32%). However, when the SDS concentration was further increased from 0.05 wt% to 0.1 wt%, the induction time rose from 2 min to 10 min. This increase was accompanied by a decrease in the hydrate conversion rate, which dropped from 23.32% to 15.28%. Kang et al. found the optimum concentration of SDS to be 100 ppm for promoting the kinetics of CO_2_ hydrate formation at 2–3.5 MPa and 273-275 K; above the aforementioned concentration, SDS acts as a kinetic inhibitor instead. Despite their advantages, surfactants also face challenges associated with their toxicity, which renders these KHPs unsuitable for certain hydrate-based applications such as HBD^22^. Additionally, foam formation during hydrate dissociation can persist for several hours^174^ posing technical and operating difficulties in utilizing these chemical substances^22^. Recently, non-ionic surfactant, such as Span and Tween, are favored for their superior stability, formulation flexibility, and biodegradability, with their inertness reducing ionic interference in diverse fluid systems and thereby supporting their application in hydrate-based processes, as either KHPs or KHIs^166,175^. Pan et al.^176^ confirmed the efficiency of Span-80 and Tween-80 as KHPs in a 40:60 water–diesel emulsion, enhancing CH_4_ hydrate formation by shortening reaction times and improving gas storage density. Furthermore, Sun et al.^177^ proposed a novel desalination approach using dispersed hydrate formation with Span 80, which, through the formation of a micron emulsion, improved CP dispersion in saline solutions, accelerated hydrate formation to yield a maximum water recovery of 92.8%, and effectively treated high-concentration wastewater, achieving a water yield of 40.2% and a removal efficiency of 86.0% in a 6.5 wt% NaCl solution, while also exhibiting broad pH tolerance, self-separation recovery, and cycling stability.

#### Amino acids

The building blocks of proteins, are non-toxic, biodegradable, economical, and environmentally friendly molecules characterized by a basic amino group (-NH_2_), an acidic carboxyl group (-COOH), and an organic R group (or side chain)^73,103,174,178^. These compounds can be categorized into hydrophobic, polar, or charged groups, based on the nature of their side chains and their interactions with polar solvents such as water^179^. These properties make amino acids suitable candidates for various industrial applications. Despite their advantages, the recovery of amino acid promoters from the resulting water is difficult and sensitive to temperature, often leading to thermal degradation^22^. Amino acids, specifically those possessing aromatic side chains and hydrophobic characteristics, have demonstrated significant potential as KHPs facilitating formation of porous hydrates. Their intrinsic high surface activity enhances adsorption efficiency onto the surfaces of forming hydrate crystals, function similarly to dispersants during hydrate formation. This effective facilitates capillary actions, which in turn promote accelerated hydrate growth rates. Cai et al.^180^ first reported L-methionine as the most effective promoter to enhance CO_2_ uptake kinetics in its hydrate formation at a concentration of 0.2 wt%, achieving a t_90_ (time required to achieve 90% of the total gas uptake) of 15 min without the use of energy-intensive mixing technologies or environmentally harmful chemicals. Rehman et al.^21^ confirmed the effectiveness of L-methionine, which exhibited the best kinetics and the most stable CO_2_ hydrates. This included the highest CO_2_ hydrate storage capacity, a 93% gas-to-hydrate conversion ratio. Shen et al.^181^ proposed a mechanism for the promotion of CO_2_ hydrate formation by L-methionine. Based on their proposed mechanism, due to their amphiphilic nature, some L-methionine molecules dissolve in the solution and others arrange themselves at the CO_2_-water interface, with hydrophilic heads submerged in water and hydrophobic tails oriented towards the gas phase. This results in the formation of hydrophobic pockets and local water ordering, similar to hydrate cages. At low concentrations, L-methionine primarily resides at the CO_2_-water interface, where hydrophilic heads adsorb onto the hydrate crystal surface, leading to steric hindrance and low tangential growth rates of CO_2_ hydrate. As L-methionine concentration increases, hydrophobic tails adsorb onto hydrate cages, reducing surface tension and promoting water transport. However, beyond the critical micelle concentration (0.05 wt%), L-methionine cannot further decrease surface tension, leading to steric hindrance and a transition to three-dimensional porous hydrate growth. Notably, when NaCl is introduced, its high ionic strength forms hydration shells around Na^+^ and Cl^-^ ions, competing with L-methionine for water molecules and significantly decreasing the tangential growth rate of CO_2_ hydrate. On another note, Liu et al.^182^ found that 0.5 wt% leucine was the most effective natural amino acid promoter for CH_4_ hydrates, achieving 90% gas consumption in 20 min without foam formation. Their study emphasized the importance of amino acid side chains, particularly aromatic ones, in enhancing hydrate formation. Combining hydrophobic and aromatic side chains significantly improved hydrate formation, especially for CH_4_ hydrates, due to their composition dependency^73,182^. However, despite leucine’s ability to act as a KHP for CO_2_ and CH_4_ hydrate formations, it inhibits the formation of C_2_H_6_ and THF hydrates^182–184^. Another example of this dual behavior is histidine, which serves as a KHP during the formation CH_4_ hydrate^185^ but inhibits CO_2_ hydrate formation^186^. Therefore, the same amino acid may exert different effects on different guest formers. To select the appropriate amino acid to act as either inhibitor or promoter, it is crucial to consider the interaction of amino acids with hydrate formers^186^, as their innate nature particularly alters within hydrate systems of hydrocarbons^35^. This dual behavior of amino acids with different hydrate formers suggests that their functionality is influenced by factors beyond just surface activity. Moreover, the side-chain length, hydropathy index, and the concentration of amino acids in solution are factors that contribute to their dual behavior^35,182^. Regarding the concentration of amino acids, it is important to note that each gas system has an optimal concentration for amino acids. Beyond this optimal concentration, their promotive or inhibitory effects diminish.

#### Nanoparticles

Various materials such as metallic-based nanofluids and carbonaceous nanomaterials, exhibit at least one dimension within the range of 1 to 100 nm. Nanoparticles offer an increased gas-liquid contact surface area owing to their large specific surface area and high surface activity, thus significantly enhancing mass and heat transfer, gas consumption, storage capacity, and water-to-hydrate conversion. The Brownian motion of nanoparticles in the fluid assists in mixing, similar to a stirrer, enhancing the driving force and reducing film resistance at the gas-water interface^73^. The shape of nanoparticles plays a critical role regarding their promoting behavior, impacting both the induction time and the amount of gas trapped within hydrate crystals^73^. Considering that hydrate formation is an exothermic process, these properties position nanoparticles as highly suitable candidates for KHPs, particularly for hydrophobic gases such as CH_4_ compared to soluble gases like CO_2_^22,73,187,188^. An important consideration in hydrate formation is the dual behavior of nanoparticles, which can either promote or inhibit the process, depending on the amount, properties, and conditions of the particles used. Cheng et al.^189^ reported that SiO_2_ nanofluids at concentrations of 0.1–0.3 wt % acted as KHP, with an optimal concentration of 0.2 wt% reducing induction time by 75.7%. However, at a higher concentration of 0.4 wt%, the same nanofluids markedly inhibited hydrate formation. Similarly, Sun et al.^190^ found that the expected value and variance of induction time, influenced by particle sizes and mass concentrations, determined whether SiO_2_ nanoparticles promote or inhibit THF hydrate formation. Therefore, when selecting nanoparticles for hydrate-based processes, particularly HBD, it is crucial to consider this dual behavior and choose the most appropriate type and amount of nanoparticles based on the specific hydrate formation conditions and formers used in order to accelerate hydrate formation.

Lu et al.^191^ proposed a plausible mechanism through the application of a graphite nanofluid as an effective KHP. According to this mechanism, upon the dissolution of CH_4_, a substantial number of CH_4_ molecules adsorbed onto the surface of graphite nanoparticles. As the solution approached CH_4_ super-saturation, the Brownian motion of particles facilitated the rapid nucleation of hydrates at the liquid-graphite interfaces. Consequently, CH_4_ hydrate films surrounding the graphite nanoparticles increased in thickness, ultimately encapsulating the graphite nanoparticles. Based on experimental investigations, this phenomenon has been shown to expedite hydrate formation, resulting in an up to 89% reduction in induction time. The tendency of metallic nanoparticles to precipitate and agglomerate, due to their high density, alters their hydrodynamic size and morphology. This results in poor dispersion and unstable colloidal systems, ultimately reducing thermal conductivity, hindering continuous heat removal, and negatively impacting the overall hydration process^73,192^. Hence, it is essential that, among nanoparticles, repulsive forces dominate over attractive forces to prevent coagulation and ensure the desired dispersion and stability. Methods such as magnetic fields, sonication, chemical additives, particularly surfactants, and modification are useful when the nanofluid is a polar solvent^193–197^. The efficiency of magnetic fields in enhancing the functionality of Fe_3_O_4_ nanoparticles for seawater desalination has also been investigated^198^, and confirmed its enhancing effects, illustrating a reduction in induction time by 89%, 22%, and 92% with the presence of 0.07 wt% Fe_3_O_4_ nanoparticles, a magnetic field, and their combined use, respectively. Additionally, the influence of SDS on various nanoparticles, such as Ag^199^, Al_2_O_3_^99^, ZnO^200^, and CuO^201^, has been investigated, illustrating its dual functionality as both a stabilizer and promoter. Pahlavanzadeh et al.^202^ stated that, although SiO_2_, Al_2_O_3_, and CuO nanoparticles significantly reduced the induction time, SDS was more effective in increasing the amount of gas consumed and the apparent rate constant of hydrate formation during the hydrate growth stage. Hence, nanoparticles enhance hydrate nucleation by increasing nucleation sites and promoting heterogeneous nucleation, while surfactants improve hydrate growth rate by reducing surface tension and altering hydrate morphology. In another study, Wu et al.,^203^ confirmed the high efficiency of Fe_3_O_4_ coated with a sodium oleate and SDS bilayer surfactant, reporting a 62% and 82% reduction in the total hydration period and the induction time, respectively, at an a concentration of 0.10 wt%, compared with pure SDS for promoting CH_4_ hydrate formation. Additionally, they stated that the use of a magnetic field could even make the induction period almost negligible.

#### Micro-nanobubbles (MNBs)

These are defined as gaseous cavities with diameters less than 1 μm, typically around 200 nm or less^204–206^. MNBs can be classified as surface MNBs, which are generated at solid-liquid interfaces, and bulk MNBs, which form within bulk liquids^207–210^. Surface MNBs exhibit a spherical cap shape with diameters of 10 to 50 nm^211^. Practical methods for the formation of MNBs include cavitation, which is the main and traditional approach, along with nanopore membranes, gas hydrate dissociation, and sonochemistry^212–221^. MNBs generated by different methods can exhibit variations in size, surface charge, longevity, and stability. Besides the generation method, various factors influence MNB size, shape, and characteristics, including the gas type and concentration^216,222,223^, sonication time^207^, solution properties, particularly pH values^224,225^, salt ion concentration^224,226^, fatty acids^227^, solid-surfaces^228^, temperature and pressure conditions^229–231^, and external electric field^222,232^. Understanding the effects of the above is crucial to optimize their performance in hydrate-based applications.

MNBs possess distinctive physicochemical properties, including large specific surface area, slow rising velocity, high internal pressure, elevated gas density, high interface potential, high mass transfer efficiency, enhanced reactivity, and notable stability in aqueous environments. The functional efficacy of MNBs is significantly influenced by their lifetime. Although theoretical calculations suggest an extremely short lifespan for MNBs (e.g., ~0.41 μs for 88.5 nm MNBs^208^), experimental evidence indicates their stability in aqueous solutions can extend over weeks or even months^233–238^. Macro-bubbles rapidly ascend to the gas-liquid interface and burst, whereas MNBs gradually decrease in size due to prolonged stagnation and the dissolution of internal gases into the surrounding water. Furthermore, MNBs are negatively charged in aqueous environments, generating repulsive forces that prevent coalescence. This electrically charged liquid-gas interface forms an electric double layer, inhibiting gas diffusion and bubble agglomeration^239,240^. Montazeri et al.^239^ investigated the stability of air MNBs under different environmental conditions and temperatures, concluding that the stability of air MNBs at low temperatures exhibits a non-monotonic relationship influenced by water self-ionization and ion mobility. Their study highlighted that MNBs remained in suspension in the presence of various chemicals at pH levels between 4 and 9, retaining a negative charge for up to 2 months. The study found that MNBs were more stable in alkaline solutions and with low concentrations of dissolved salts, while higher concentrations led to coalescence and increased size of MNBs. These attributes underpin the potential of MNBs for various large-scale applications^241–249^, ranging from drinking water treatment^250,251^ and wastewater management^251–253^, to ecosystem restoration^254,255^, fuels combustion^256^, mineral processing^257,258^, surface cleaning^206,259^, oxygenation in agriculture, aquaculture, and disinfection^255,260,261^, to medical uses^262,263^.

Recent studies have highlighted the potential of MNBs as KHPs in HBD processes, enhancing hydrate nucleation due to their prolonged existence, extensive gas-liquid interface, high mass transfer efficiency, ability to increase gas aggregation saturation within solutions, and capacity to form hydrophobic surfaces^25,230,264–267^. Optimizing bubble sizes to maximize the gas-liquid interface area further intensifies mass transfer processes, thereby improving the efficiency of these of these KHPs in HBD^268,269^. Therefore, there is significant potential when MNBs are present during the hydrate formation stage, serving as nucleation sites, promoting gas hydrate nucleation and reducing the induction time^224,267,270–272^. Uchida et al.^267^ found that MNBs act as heterogenous nucleation sites, significantly reducing the induction time from 23.95 min in pure water to 4.49 min in the presence of C_2_H_6_ MNBs. Interestingly, ultrasound was found to promote this positive impact, by increasing the gas solubility, thus overall promoting hydrate formation kinetics. Liu et al.,^272^ investigated the impact of ultrasonic waves on MNB generation and found that increased ultrasound duration and power elevated MNBs concentration, which reduced hydrate formation induction time by up to 61.13%. Li et al.^273^ proposed a memory effect hypothesis based on MNBs, showing that guest molecules from hydrate dissociation accumulate as MNBs, facilitating nucleation during hydrate reformation. In another study, Feng et al.^217^ corroborated these findings, demonstrating that MNBs shortened the average induction time by 27.48%, increased nucleation probability by 50%, and reduced growth time by 60% compared to deionized water. Finally, Montazeri et al.^25^ utilized CO_2_ MNBs as KHPs, emphasizing their dual advantages: CO_2_ gas inclusion and the elimination of separation steps from the resulting water. Their findings demonstrated that the presence of CO_2_ MNBs in solution resulted in an 86% reduction in induction time and in accelerated water recovery rates by 69% and 63% for 0.5 M NaCl and synthesized seawater, respectively. This efficiency underscores the potential of MNBs as green and efficient KHPs, suggesting a promising future for the commercialization of MNB-boosted HBD processes.

The promoting behavior of MNBs has been generally attributed to the memory effect^274–277^, suggesting that, aside from enhancing nucleation sites and reducing the induction time, identical guest molecules in gas hydrates and bubbles release guest molecules upon bursting, thus promoting hydrate growth. During hydrate dissociation, released gas molecules cause supersaturation, forming bubbles around hydrate crystals. Consequently, MNBs enhance hydrate formation, reduce induction time, and accelerate growth rates, establishing themselves as promising KHPs. Nevertheless, some studies support the gas dissolution hypothesis^37,278^ as an explanation for MNBs’ promoting behavior. Regarding this context, Uchida et al.^271^ used C_3_H_8_ hydrate to investigate the kinetic promotion effects of MNBs. Their reports illustrated that MNBs, both formed after hydrate dissociation and prepared with an MNB generator, increased the nucleation probability by 1.3 times within 50 h compared to pure water and shortened the induction time by nearly half. They concluded that MNBs of hydrate formers mainly accelerate the hydrate formation process, while the memory effect plays a minor role by simply helping MNBs remain present in the system. Thus, the gas dissolution hypothesis is the main explanation for this promotion. MNBs play a crucial role in inhibiting hydrate decomposition^167,279,280^, as highlighted by Guo et al.^281^ through the MNB inhibition mechanism. Hydrate dissociation releases the gas formers into the liquid phase causing supersaturation. If gas diffusion out of the liquid phase is insufficient, gas molecules agglomerate and form MNBs. These MNBs, encapsulated within the hydrate, gradually release gas formers during hydrate decomposition, increasing local gas concentrations near the MNB-hydrate interface. This promotes microstructure formation necessary for hydrate nucleation, enhancing the driving force toward nucleation and consequently inhibiting hydrate decomposition. Besides, the presence of MNBs around dissociated hydrates, coupled with the memory effect, significantly enhances hydrate reformation kinetics^230,282^. Table 2 summarizes several investigations on the application of hydrate-based technologies in the desalination of various feed waters, highlighting their potential through the use of different enhancing methods, such as THPs and KHPs, as well as other innovative substances or apparatus.

**Table 2:** Comprehensive summary of HBD studies: key parameters, efficiency metrics, and operational insights

Feed water	Hydrate Former	Promoters (THPs and KHPs)	Solid-liquid separation	Salt removal efficiency	Pressure (MPa)	Temperature (K)	Water conversion	Remarks	Ref.
3 wt% NaCl	CP (0.9–2.3 mol.%)	-	Vacuum	70–90%	0.1	<279.6 K ΔT = 5.6 K ΔT = 3.6 K	15–40%**	**At 5.6 K, the removal efficiency for NaCl ranges from 89–73% at low CP concentrations, resulting in 15–20% water conversion. **With a hydrate former concentration of 2.3 and 40% theoretical water conversion at 5.6 K, the removal efficiency declines. ** At 3.6 K, salt removal ranges from 87% to 67%, with optimal results at 15% water conversion.	^15^
8.95 wt% NaCl	CO_2_	-	Vacuum	74%	3.1	Various temperatures	-	-Depending on the temperature, hydrates may not form or may form within an induction time ranging from 0 to 23.5 min	^54^
	CO_2_	CP		91%	3.1		-	-Induction time: 0	
	CO_2_	CH		95%	3.1		-	- Induction time: 0, or hydrates might not form depending on the temperature.	
3.5 wt% NaCl	CP (3 mol%)	-	Vacuum with additional steps	9–98%	0.1	274, 277	9%-72%	- Centrifuging achieved a 96% average salt removal efficiency but is costly and challenging for large-scale feed water treatment. - Combining effective mechanical methods with washing techniques is likely to maximize efficiency. - Freshwater washing improved salt removal efficiency to 93%. - Sweating reduced salt on the crystal surface by over 95% but also proportionally decreased water production, requiring optimization of the duration.	^347^
Solutions with 0.01–0.6 mol/L concentrations of lithium salts	CP	Graphite powder (GPs)	Vacuum + Centrifuge	Single-stage: 66–82.91% Two-stage: 97.41% Three-stage or more: >99.00%	0.1	275	35%–83%	-Halide ions hindered CP HBD, with an order of Cl^− ^< Br^− ^< I^−^ - Induction time mitigated from 7 days to 30 min via addition of GPs.	^33^
Seawater	CO_2_/C_3_H_8_ (90/10 mol.%)	C_3_H_8_	Washing + Purging	80–85%	Initial pressure: 2	278	27%	- A novel multifunctional desalination apparatus.	^58^
Produced water (TDS levels reaching up to 171,000 mg/l)	-CO_2_ -Natural gas	-	Third stage separating container (rinsing with fresh water)	43–86%	-3.5 -9.5	274.2	-	- Desalting efficiency CO_2_ is higher than natural gas.	^364^
Produced water (TDS levels reaching up to 171,000 mg/l)	CO_2_	-	Washing	82–89.2%	3.5	274.2	-	- Applicable for produced water with TDS less than 160,000 mg/L	^55^
Caspian seawater	THF	-	Filtration	40–80%	6	Cooled-surface temperature = T_0_ = 273.1-280.3	-	-	^57^
Seawater	CP	Graphite Powder	Vacuum + Centrifuging	71–86%	0.1	275	38–56%	- The induction time greatly shortened from 72 h into 3 h in the presence of GPs. - Stirring under 600 rpm as well as introduction of GPs enhanced salt rejection.	^365^
1–8 wt% NaCl	R410a	CP	Washing	68–86.9%	0.9–1.1	275.15	58–77%	-0.9 MPa pressure and in the presence of CP presented the best performance on ion removal efficiency. - Very short induction time and nucleation at the initial min.	^366^
Seawater	CO_2_	CP	Squeezing	56–86%	2.5	277.15	-	- The order of cations in removal efficiency: Na^+^ (86%) å K^+^ (84%) å Mg^2+^ (81%) å Ca^2+^ (78%). - The anion removal efficiency’s order: SO_4_^2-^ (62%) å Cl^-^ (56%).	^219^
Produced water	Compressed natural gas	-	Washing	79–84%	9.5	274.2	-	- Applicable for produced water with TDS less than 160,000 mg/L - Salinity of produced water can be reduced by about 82%, and 79.5-82% of cations, and 81-84.3% of anions.	^16^
3 wt% NaCl	CO_2_/C_3_H_8_ (90/10 mol.%)	C_3_H_8_	Filtration	72–89%	2.6	274.2	34–39%	- Utilized liquefied natural gas (LNG) cold energy - Induction time varies from 0.33 to 4.67 min in a cylindrical annular bed and from 0.33 to 24.33 min in a cylindrical core bed.	^338^
3.3 wt% NaCl	CH_4_/C_2_H_6_ (75/25 mol%)	-	Washing	45–80%	Initial pressure: 4.2	- Initial temperature: 274.6, ΔT = 7–12.	-	- Induction time varies from 0 to more than 600 min at various subcooling and replenish time.	^350^
0.17–5 wt % NaCl	CP	-	Gravity + Filtration + Washing	81%	0.1	271, 273, and 275	4–35%	-	^285^
3–5 wt% NaCl	CP	-	Vacuum filtration + Washing	80–90%	0.1	274.1–277.1	23–97% (at 274.1–277.1 K and 300 rpm)	- Increasing stirring, promoted hydrate formation but adversely affect the removal efficiency. - Excess CP increase the yield of dissociated water while the removal efficiency stayed around 80%. - The existence of an inverse correlation between removal efficiency and water conversion. - Higher temperatures led to smaller yield and higher efficiency. - Washing led to an increase in removal efficiency of ions from 50.5% to over 79.0%.	^3^
Solution with 3.4 wt% salinity	CP (3 mol%)	-	Washing + Vacuum	54–90% (Single stage with filtration = about 63% With washing + filtration step = Up to 90%)	0.1	277.15	41.2–53.3%	successive washing treatments as solution upon the drop of removal efficiency after reaching the theoretical limit of water-to-hydrate conversion.	^348^
Heavy metals containing solutions (93.36–140.4 mg/L)	R141b	-	Washing + vacuum + centrifuging	88.0–90.8% (Without-washing: 50.1–71.8% With-washing: 73.1–99.9%)	0.1	277	61.7–80.0%	Removal efficiency (RE) with washing step: - Cr^+3^: 140.04 mg/L → RE: 99.85% - Cu^+2^: 143.9 mg/L → RE: 99.75% - Ni^+2^: 140.04 mg/L → RE: 99.85% - Zn^+2^: 140.04 mg/L → RE: 73.13%	^64^
Seawater with a TDS concentration of 32,294 with multiple-nuclides.	HFC-134a	-	Partial melting + Washing	79.5–98.5%	0.28	274.7	About 15% (in 55 min)	-Ion removal efficiency ranging from 98.9% to 99.2%, with an order of I^-^ > Cs^+^ > Co^2+^ > Sr^2+^.	^367^
Industrial Effluent	HFC134a/ C_3_H_8_ (50/50 mol.%)	-		80–95%	0.6	278.3	<33.6%	- Induction time varying from 3.92 to 6.33 min - Hydrate formation rate: 0.51-0.75 cm^3^/min	^368^
	C_3_H_8_ (100 mol.%)	CP (6 mol.%)		47–60%	0.3	278.0	<34.5%	- Induction time varying from 4.58 to 6.75 min - Hydrate formation rate: 0.52-0.83 cm^3^/min	
Seawater	CO_2_/C_3_H_8_ (90/10 mol.%)	C_3_H_8_	Gravity + purging + Washing	80–86%	2	278	25.5–27%	- The effluent liquids after 10 min in hydrate-purging process was regarded as freshwater, selecting it as the optimal separation method. -The removal efficiency is related to the strength of ionic hydration. - Freshwater recovery > 30%	^369^
Effluents containing K_3_PO_4_	CP	Graphite Powder	Filtration + centrifuging + washing	54–88%	0.1	271–276	-	- Water recovery declines with temperature. - The time-dependent water recovery shows a three-stage feature, including exponential growth, linear increase, and stable stages, owing to the interaction between the driving force of supercooling and the increasing K_3_PO_4_ concentration during the reaction processes. - water recovery = 18–85%	^370^
Saline water (1.0–3.5 wt%)	R134a/CP	CP	Washing + Centrifuge	45–75%	0.1	275.15	å 60%	- After a single-stage hydrate treatment, the removal efficiencies for ions and TDS ranged from 74.1% to 82.8% and from 70.3% to 72.9%%, respectively.	^371^
Saline solution	CO_2_	CO_2_ Nanobubbles	Vacuum filtration	35–48%	3.58	274.15	12.3–93.1% (from 30 min to 180 min)	- NBs reduce the induction time from 43 to 6 min, compared to the pure water. -Water recovery from 5% to 69%, higher than the typical water recovery achieved by RO. -Ion removal efficiency 40–50% in a 3-h HBD single-stage process of seawater.	^25^
3.4 wt% NaCl	CO_2_ + C_3_H_8_	Graphite porous media/CP	-	-	2.0–3.0	275.6	35.62 (±1.76) % to 43.25 (±0.14) %.	- Combination of CP and graphite reduced the hydrate induction time from 8.25 (±4.42) mins to 1.95 (±1.7) mins -CP can act as a dual functional promoter. - Optimal CP/water volume ratio is 0.175. - Too much graphite and hydrate crystals may accumulate at the gas–liquid interface and prevent hydrate formation.	^372^
Produced water	CO_2_ + C_3_H_8_	C_3_H_8_	-	47–63%	2.0	275.15 and 277.15	24.03% at 275.15 K 21.39% at 277.15 K	- The induction time for produced water at 277.15 is 138.49 mins, while at 275.15 is 53.83 mins.- The induction time increased by 52.64% in treating 2.8 wt% and a decrease in moles of gas consumed, water recovery, and water-to-hydrate conversion by 6.99%, 2.08%, and 9.69% compared to the deionized water system at 275.15 K.^-^ Cation removal order K^+^ > Na^+^ > Mg^2+^ > Ca^2+^ at 275.15 K.- Anions removal order: SO_4_^2− ^> Cl^−^ at 275.15 K	^373^
1–3.5 wt% NaCl.	Compressed natural gas (CNG)/CP	CP(0, 0.5, and 1 mol.%)	-	0–83%	6.5, 8, and 9	274.15 and 276.15	-	The highest removal efficiency was 83.13% for a 1 wt% NaCl solution in the presence of CP at 274.15 K, while the lowest salt rejection efficiency was 56% for a 3.5 wt% NaCl solution in the absence of CP at 276.15 K.	^374^
Produced water	CO_2_	-	using a novel filter-based apparatus + Washing (with deionized water)	62–80%	2.5, 3, and 3.5	275.15, 277.15	<39%	- A novel filter-based hydrate desalination reactor - At optimal conditions (500 ml, 3.0 MPa, 450 rpm, 275.15 K), the induction time, moles consumed, water-to-hydrate conversion, and water recovery were 57 mins, 1.28 mol, 27.72%, and 50.65%, respectively. - Induction time: 54.58 mins at 275.15 K and 66.99 mins at 277.15 K. Induction time: 107.22 mins at 350 rpm and 96.65 mins at 400 rpm.	^375^
Radioactive wastewater	CO_2_	-	Filter papers + Squeezing	96–99.9% (based on the initial concentrations of Cs^+^ and Sr^2+^)	2.5 ??	273–284	-	- The CO_2_ HBD can effectively treat radioactive wastewater containing Cs^+^ and Sr^2+^ across a broad concentration range with comparable recovery efficiency. - The initial formation rate varied from 3.06 for pure water to 1.35 for 10 wt% SrCl_2_.	^376^
Hypersaline brine (8.0 wt% NaCl)	HFC-125a,	-	Squeezing + Pre-melting + Squeezing	75–99% (from 0 to 80% induced-melting)	0.25	272.65 (ΔT = 2.4, 4.3, and 7.8 K)	12% (in 2.64 h)	- HFC-134a and HFC-152a exhibit rapid gas uptake kinetics, whereas HFC-125a shows sluggish kinetics. - A novel concept of hydrate palletization using induced melting was utilized to enhance removal efficiency. - Induced hydrate pellet melting significantly improved salt removal efficiency, showing a non-linear behavior where the increase rate slows and approaches a maximum asymptotically as more hydrate melts.	^20^
	HFC-134a			74–97.2% (from 0 to 80% induced-melting)			12% (in 0.73 h)		
	HFC-152a			75–99.7% (from 0 to 80% induced-melting)			12% (in 0.96 h)		
3.3 wt% NaCl	CO_2_	C_3_H_8_	-	-	2.5-4	275.15–279.15	<39.5% (at 400 rpm up to 19 mol% C_3_H_8_)	- Optimal hydrate kinetics were achieved at 4 MPa and 275.15 K. - A 19% C_3_H_8_ concentration in N_2_ + C_3_H_8_ and Ar + C_3_H_8_ mixtures showed optimal hydrate formation kinetics due to enhanced phase equilibrium. However, for CO_2_ + C_3_H_8_, this ratio increased the final water conversion rate but reduced the formation rate and number of hydrates formed within 1 h. - At 400 rpm with 0 to 19 mol% C_3_H_8_, the t_90_ varied from 0.58 to 7.83 h for CO_2_, from 8.58 to 38.23 h for N_2_, and from 3.30 to 6.78 h for Ar.	^119^
	N_2_						<58.4% (at 400 rpm up to 19 mol% C_3_H_8_)		
	Ar						<42.2% (at 400 rpm up to 19 mol% C_3_H_8_)		
Hypersaline brine (10.0 wt% NaCl)	R152a (C_2_H_4_F_2_)	-	Pressing + Vacuum filtration (P-VF)	Up to 80% for ΔT = 2 K	0.2	ΔT = 2, 3, and 5 K	<30% (for ΔT = 2 K and 3 K) <40% (for ΔT = 5 K)	- R152a hydrates are stable under 0.4 MPa at temperatures between 273 K and 283 K, even with high NaCl concentrations (5.0 wt% and 10.0 wt%). - Emphasizing the potential of R152a HBD for sustainable hypersaline brine treatment. - Na^+^ ions are easier to remove than Cl^-^ ions due to lower free energy barriers. - Elevated temperatures slow growth rates but increase removal efficiency.	^304^
			Washing + Vacuum filtration (W-VF)	Up to 72% for ΔT = 2 K.					

### Separation, dissociation, and recovery

Utilizing appropriate substances and infrastructure under favorable conditions facilitates hydrate formation. Post-formation, hydrates must be separated from the resulting brine, which has higher salt and ionic concentrations than the initial feed water, using gravity or mechanical separation methods such as centrifugation, filtration, sedimentation, and flotation. These methods can be combined, for example, with vacuum filtration and centrifugation to enhance separation efficiency. Coupling these steps with a wash column can remove excess salt from the harvested hydrates. However, washing steps, while producing clean water, can reduce overall efficiency and increase processing costs^17,32^. After separation, the next step is to dissociate the hydrates to obtain clean water devoid of salt, ion, and other unwanted impurities. Hydrate dissociation is a process that involves breaking various bonds, including hydrogen-bonds and van der Waals forces, within the hydrate lattice. Several methods can be used to dissociate hydrates, including depressurization, thermal stimulation, electrical stimulation, microwave irradiation, and ultrasound, or a combination of these methods. Each method has its own advantages and disadvantages, and the choice depends on the specific application and the properties of the hydrate solids and brine solution. The recovery of clean water is the primary outcome of these steps in the HBD process.

Materials extracted from the hydrates, such as hydrate formers and promoters, should be recovered and recycled to sustain the process’s sustainability. Recent studies have shown that the use of efficient, economical, and green hydrate formers and promoter molecules can significantly enhance the formation and dissociation of gas hydrates and reduce the energy consumption and overall cost of the process^165,283,284^. These advancements introduce them as promising sustainable chemical additives in HBD processes. For example, since for selecting hydrate formers, a critical factor is the feasibility of water separation from the formers, immiscible liquid hydrate formers such as CP^3,285,286^ and TBAB^287,288^ have emerged as a promising option due to their lower operating expenses and ease of separation through centrifugation and washing. Additionally, incorporating MNBs as a chemical component not only accelerates the hydrate formation rate, but also significantly reduces overall operational costs. This reduction is primarily due to their ability to bypass additional separation steps, as they seamlessly integrate into the process without leaving residual components requiring removal from the resulting water^25^. Consequently, MNBs deliver both performance improvements and economic advantages, making them an effective choice for process optimization. Furthermore, integrating environmentally friendly and biodegradable hydrate formers like TBAB and CP can reduce the environmental footprint of the hydrate-based processes. Regarding chemical promoters, recyclable magnetic Fe_3_O_4_ nanoparticles coated with SDS^102^ not only exhibited higher efficiency than non-coated Fe_3_O_4_ in reducing induction and reaction times but also demonstrated high recyclability and cost-effectiveness, allowing for multiple uses in hydrate formation. Overall, the strategic recovery and recycling of both hydrate formers and promoters not only bolster the sustainability of gas hydrate processes but also pave the way for cost-effective and environmentally benign advancements in the field.

## Environmental and safety concerns

The environmental impact of desalination methods is a critical benchmark for technology selection, with global warming potential (GWP), serving as a key indicator. GWP quantifies the energy absorption over a specified time horizon, typically 100 years, resulting from the emission of one metric ton of a gas relative to that of one metric ton of CO_2_. A higher GWP indicates that the gas contributes proportionally more to global warming during this period compared to CO_2_. Conventional technologies such as MSF and MED exhibit high GWP values (23.41 and 18.05 kg CO₂ eq/ m^3^, respectively), while RO’s GWP typically ranges between 1.75 and 6.10 kg CO₂ eq/m^3^, largely driven (~70%) by its electricity consumption^289–291^. Integration of renewable energy can mitigate these impacts, although it may result in a modestly higher production cost. Moreover, brine disposal, an inevitable byproduct of desalination, further compounds environmental concerns by contributing both to marine eutrophication and to an increased effective GWP of desalination methods^292^.

Recent research has sought to reduce both energy consumption and environmental costs, sometimes by incorporating chemical additives. However, as Lee et al.^293^ noted, increased chemical usage to lower energy requirements can introduce additional environmental burdens, quantified as environmental cost (EC) values ranging from 0.16 to 0.50 USD/m^3^ ^294–296^. In this context, HBD emerges as a promising novel technology, while its own life cycle assessment (LCA) and environmental life cycle cost (ELCC) analysis have not been investigated comprehensively yet. Lee et al.^290^ investigated these items in an integrating HBD with RO process using C_3_H_8_ as the hydrate former. Their analysis demonstrated that depending on the feedstock used for production of C_3_H_8_, and the handling of renewable energy certificates (RECs), this hybrid system could achieve a GWP as low as 0.016 kg CO_2_ eq/m^3^ when waste cooking oil is used as the feedstock for C_3_H_8_ and REC sales are excluded. Economically, the production cost was competitive, ranging from 2.29 to 2.86 USD/m^3^, with profitability observed across all configurations. Additionally, their investigation highlighted that freshwater ecotoxicity was predominantly driven by the release of copper and zinc ions during natural gas production, while marine ecotoxicity was mainly associated with brine discharge, primarily due to the presence of highly toxic metals such as silver. Overall, this study underscored that while the RO-HBD hybrid approach reduces global warming impacts through the utilization of LNG cold energy and electricity generation, a trade-off exists between economic feasibility and environmental sustainability. Optimizing hydrate former selection, mitigating chemical impacts, recycling, and addressing residual environmental concerns, particularly regarding byproduct disposal, are essential for advancing HBD as a sustainable alternative in desalination. Future research should strive to further elucidate the environmental life cycle impacts HBD to comprehensively assess its potential as a competitive desalination method. In this section, we critically examine the environmental consequences and toxicity concerns associated with HBD.

Environmental implications constitute a significant concern with HBD, largely due to the reliance on chemical additives, specifically, hydrate formers and promoters, that may exacerbate global warming, contribute to ozone depletion, and result in the persistent accumulation of harmful substances. Early investigations evaluated ostensibly non-toxic, highly water-immiscible compounds, namely, chlorofluorocarbons (CFCs), hydrofluorocarbons (HFCs), and hydrochlorofluorocarbons (HCFCs), as hydrate formers; however, their inherent flammability, significant greenhouse effects, and ozone-depleting properties have severely limited their applicability in HBD^30,59,297^. This prompted the pursuit of more environmentally sustainable alternatives, such as CO_2_ and C_3_H_8_, which emerged as attractive candidates for HBD. Although CO_2_ is relatively non-toxic and odorless, it reacts chemically with water and remains a significant greenhouse gas; similarly, C_3_H_8_ is largely inert and non-toxic under standard conditions, albeit with the caveat of its flammability^7,31^. These characteristics position both gases as promising hydrate formers, provided that their intrinsic chemical behaviors and associated risks are carefully managed^298,299^. Additionally, natural gas, owing to its widespread availability, non-toxic nature, and mild hydrate formation conditions, is regarded as an environmentally sustainable hydrate former^59,300^. Notably, the strategic selection of low-GWP gases as hydrate formers is essential^301^. For instance, R152a not only exhibits milder hydrate-forming conditions compared to R134a^302,303^, but also has a substantially lower GWP, 124 versus 1430 for R134a^304–306^. While CH_4_ exhibits a GWP ~25 times that of CO_2_ and an atmospheric lifetime of 12 years, SF_6_ is recognized as the most potent greenhouse gas, with a GWP 22,800 times that of CO_2_ and an atmospheric lifetime of ~3200 years^306^. The incorporation of low-GWP gases minimizes the environmental impact of unintended emissions during hydrate formation and dissociation, ensuring that any gas release contributes minimally to climate change. Moreover, this approach aligns with evolving environmental regulations and sustainable process design, thereby enhancing the overall viability and long-term sustainability of hydrate-based technologies. As a result, adopting low-GWP gases not only reduces the carbon footprint of these systems but also supports their broader application in desalination and wastewater treatment, contributing to more environmentally responsible industrial practices. Table 3 summarizes the lifetimes and GWPs of several practical gas molecules employed as hydrate formers, thereby offering a useful framework for their informed selection and management to mitigate long-term environmental impacts. Consequently, inaccurate selection of hydrate formers can jeopardize environmental safety by increasing greenhouse gas emissions and posing risks to human health, ultimately impeding the widespread application and commercialization of HBD technologies.

**Table 3:** Atmospheric lifetimes and GWPs of common gas hydrate formers^306^

Chemical compound	Chemical formula	Lifetime (years)	GWP (100 years)
Carbon dioxide	CO_2_	See below^a^	1
Methane	CH_4_	12	25
HFC-141b	CH_3_CCl_2_F	9.3	725
HFC-134a	CH_2_FCF_3_	14	1,430
HFC-152a	CH_3_CHF_2_	1.4	124
Sulfur hexafluoride	SF_6_	3,200	22,800

^a^The CO_2_ response function used in this report is based on the revised version of the Bern Carbon cycle model used in Chapter 10 of this report (Bern2.5CC; Joos et al.): using a background CO_2_ concentration value of 378 ppm. The decay of a pulse of CO_2_ with time t is given by: $C(t)={a}_{0}\mathop{\sum }\nolimits_{i=1}^{3}{a}_{i}\times {e}^{\frac{-t}{{\tau }_{i}}}$, where a_0_ = 0.217, a1 = 0.259, a_2_ = 0.338, a_3_ = 0.186, τ_1_ = 172.9 years, τ_2_ = 18.51 years, and τ_3_ = 1.186 years.

Furthermore, while incorporating THPs and KHPs substantially reduces the intrinsic kinetic limitations and high energy demands associated with hydrate formation, their use often faces challenges associated with environmental and safety concerns, particularly toxicity, foam formation, persistence, and bioaccumulation risks. For example, THF, a widely used THP^103,307,308^, is highly flammable and classified as hazardous under the Globally Harmonized System^309^. While exhibiting low to moderate acute toxicity (LD_50_: 1650 mg/kg in rats) with minimal aquatic risks, its volatility and water miscibility elevate environmental mobility and exposure concerns^310–313^. Interestingly, THF is inherently biodegradable, highly volatilizable, and has low bioaccumulation potential, reducing long-term ecotoxicity concerns^310,311^. Regulatory agencies, including the U.S. EPA and the European Chemicals Agency (ECHA), impose strict discharge limits, such as the 8.4 mg/L threshold under U.S. pharmaceutical effluent guidelines, to ensure its controlled use and mitigate environmental and health risks^312,314^. SDS, frequently used as a KHP, exhibits slightly higher toxicity than THF (LD_50_: 1288 mg/kg in rats) and persistence in aquatic environments, contributing to bioaccumulation and microbial disruption, which complicates its disposal^63,315^. Chronic exposure has been linked to cell membrane disruption, raising concerns for both environmental and human health^174^. SDS is biodegradable under specific conditions, but its potential for long-term environmental impact, high toxicity, and foam formation during hydrate formation necessitate stringent management. Therefore, chemical selection is key to the success and overall sustainability of HBD^29,316^. Table 4 presents a comprehensive summarize of key environmental and health information for selected THPs and KHPs used in HBD. Table 4 includes data on toxicity (LD₅₀), aquatic toxicity, and legal or recommended limits in wastewater effluents, which can serve as guidelines for the permissible concentrations of these additives when used in the HBD process.

**Table 4:** Summary of toxicity profiles, aquatic impact, and regulatory discharge limits for additives in HBD

Promoter	Type	Oral LD_50_ (Rat)	Aquatic Toxicity (LC_50_)	Legal/Recommended Limit in Wastewater Effluent	References
Tetrahydrofuran (THF)	THP	1650 mg/kg	Low toxicity to aquatic organisms; inherently biodegradable; low bioaccumulation potential	8.4 mg/L (U.S. pharmaceutical effluent guidelines) *DNEL = 72.4 mg/m³	^310–313,377^
Cyclopentane (CP)	THP	11,400 mg/kg	Limited data; low water solubility suggests low aquatic toxicity; non-ozone-depleting; low global warming potential (~20) LC_50_: 106 mg/L, vapor (Rat)	Not specified DNEL _inhalation_: 1210 mg/m³ (for workers) DNEL _inhalation_: 643 mg/m³ (for general)	^378,379^
Sodium Dodecyl Sulfate (SDS)	KHP	1288 mg/kg	Low to moderate toxicity, biodegradable in some cases, high bioaccumulation potential, LC₅₀ (Pimephales promelas): 6.6 mg/L (96 h); LC₅₀ (Ceriodaphnia dubia): 48 mg/L (48 h); potential for bioaccumulation LC_50_ > 3.9 mg/L (Rat), 1 h	Not specified DNEL_inhalation_: (1210) mg/m³ **PNEC_(fresh water)_: 10.6 mg/L EU value: 1200 mg/m³	^380–383^
Span 80 (Sorbitan Monooleate)	KHP	>5000 mg/kg	Considered non-toxic to aquatic life; biodegradable; low bioaccumulation potential	Not specified	^384,385^
Ag NPs	KHP	Variable	NOEC for Hyalella azteca: 0.9 µg/L; LOEC: 1.9 µg/L; potential for bioaccumulation and chronic toxicity in aquatic organisms	Not specified	^386^

(*) DNEL stands for “Derived No-Effect Level”, while (**) PNEC refers to the “Predicted No-Effect Concentration”.

Due to the environmental and ecological risks associated with conventional hydrate promoters, research increasingly prioritizes the development of biodegradable, environmentally benign, and low-toxicity alternatives. These advancements aim to enhance the sustainability of HBD while maintaining safety and regulatory compliance, ensuring that process efficiency is achieved without exacerbating long-term environmental harm. Recent research has emphasized the development of biodegradable and environmentally benign promoter alternatives that maintain performance while reducing ecological impacts. An optimal bio-compatible KHP should not only improve the efficiency of the HBD process, but also exhibit minimal toxicity, environmental sustainability, cost-effectiveness, recoverability, high thermal conductivity, and thermal stability to mitigate ecological and public health risks.

Biodegradable amino acids, which are essential dietary components, have recently garnered attention as an effective class of KHPs^174,317^. Amino acids, such as phenylalanine, histidine, L-valine, L-cysteine, L-methionine, and L-threonine, exhibit promising attributes, including non-foaming behavior, biodegradability, and operational simplicity, although challenges related to thermal degradation and difficult recovery persist for some variants. For instance, Khan et al.^318^ demonstrated that incorporating 1 wt% tryptophan, a biodegradable amino acid, in CO_2_ hydrate systems reduced the induction time by 50.61%, increased the initial formation rate by 144.5%, enhanced water recovery by 121%, and improved gas uptake by 124%. Owing to their inherent non-toxicity, amino acids eliminate the need for separation from dissociated water^185,186,316^.

Biosurfactants, distinguished by their biodegradability, low toxicity, and tunable amphiphilicity, adhere to stringent environmental regulations and exhibit remarkable stability under extreme pH, temperature, and saline conditions. These attributes position them as promising green alternatives to conventional KHPs, facilitating gas hydrate nucleation and growth without compromising efficiency^315,319–322^. As surface-active compounds derived from microorganisms, biosurfactants enhance hydrate formation by leveraging their hydrophobic and hydrophilic domains to reduce interfacial tension, adhesion energy, and contact forces among hydrate, water, and gas molecules^315^. Rhamnolipid, an environmentally compatible biosurfactant with strong hydrophilic and hydrophobic properties, has been identified as an effective KHP for CO_2_ hydrate formation, reducing induction and total process times by 99% and 84%, respectively, compared to SDS^323^. Similarly, surfactin, a cyclic lipopeptide biosurfactant, markedly enhances CH_4_ hydrate formation relative to pure water and SDS (1 wt%), with comparative studies indicating optimal performance at 200 ppm for rhamnolipid and 400 ppm for surfactin^324,325^. However, concentrations above 400 ppm can diminish performance and potentially induce hydrate inhibition, necessitating careful assessment of both biosurfactants for inhibition applications at concentrations below their critical micelle concentration but not below 400 ppm^315,325^. Mirzakimov et al.^326^ recently reported rapid water-to-hydrate conversion using bio-based KHPs, addressing key challenges such as foam formation, hydrate stability, and environmental impact in hydrate-based technologies. Employing a green synthesis approach, they developed four novel biosurfactants (BSCOs) from castor oil, significantly enhancing CH_4_ hydrate formation. These biosurfactants demonstrated strong environmental compatibility, with 20.8% degradation over 28 days and no detectable toxicity in mice, and high hydrate stability, exhibiting only 7.5–10.5% dissociation at 1 atm and 268 K after 14 days. While surfactants are traditionally considered to improve hydrate formation kinetics without affecting thermodynamic properties, emerging studies suggest that biosurfactants may influence both. For instance, rhamnolipids, demonstrated a 42.97% increase in CH_4_ hydrate formation rate, a 22.63% reduction in induction time, and an upward shift in formation temperature with minor modifications in hydrate cavity ratios^327,328^.

Among such green additives, starch has been extensively explored in hydrate-based systems. While some earlier studies reported that various starch classes inhibit hydrate formation^329^, recent investigations have elucidated their potential as promoters. For instance, potato starch has been identified as a bio-compatible KHP for CH_4_ hydrate formation, demonstrating comparable efficacy to SDS at equivalent concentrations, attributing to phosphate groups in its molecular structure, which strengthen anionic behavior and promote water hydrogen bonding^32,330^. Similar to potato starch, an investigation into corn dextrin, a green, biodegradable, biocompatible, and water‐soluble linear polymer derived from the hydrolysis of starch, as a KHP for CH_4_ hydrate formation. This investigation illustrated that at 1 wt%, the induction time decreased drastically from 1256 to 18 min, and gas uptake kinetics improved significantly^331^. Notably, the apparent growth constant for 7 wt% dextrin was ~28 times that of pure water, indicating that corn dextrin is a competitive alternative to SDS for promoting methane hydrate formation. In another report, Alizadeh et al.^332^ investigated the effect of sucralose, a novel environmentally friendly promoter, on CO_2_ hydrate formation, reporting that at 0.75 wt% sucralose, gas consumption and hydrate storage capacity were maximized, with a 37% increase in consumption at 300 min and a 35% improvement in water-to-hydrate conversion at 20 min relative to pure water. However, concentrations above 0.75 wt% yielded diminishing returns, with slight inhibitory effects observed at 10 wt%, and elevated cell temperatures further impaired the kinetic parameters of hydrate formation.

MNB promoters offer an environmentally benign approach to gas hydrate formation by eliminating the need for chemical additives and leaving no residual contaminants in recovered water. Comprised solely of gas and water, often inert or mildly reactive gases, MNBs dissolve or effervesce post-hydrate formation, ensuring no persistent residue or adverse ecological impact^23,25,252,262^. Their integration into hydrate-based processes is seamless, requiring no additive cleanup or additional separation steps^25^. Moreover, MNBs exhibit negligible toxicity risk; for instance, CO_2_ MNB-assisted desalination demonstrated no introduction of contaminants, preserving the integrity of treated water. Unlike conventional surfactant-based promoters, MNBs prevent foam formation, facilitating clean gas release and efficient water recovery upon hydrate dissociation. In contrast, biosurfactants, though biodegradable, can induce foaming, as observed in certain coconut-derived surfactants akin to SDS^315^. Energy requirements for MNBs remain minimal, as bubble generation relies primarily on moderate pressure or shear rather than chemical activation, significantly reducing downstream pollution and disposal concerns. Their compatibility with sustainable engineering is well established, with ozone or air nanobubbles already employed in water treatment applications, reinforcing their potential as green alternatives for hydrate-based technologies^299,333^.

Advancements in green alternatives, including amino acids, biosurfactants, starch-based materials, and MNBs, underscore the potential for reducing HBD’s reliance on hazardous compounds like THF and SDS. Additionally, innovative approaches such as LNG integration promise to significantly reduce operational costs, further strengthening its feasibility. These advancements position HBD as a promising desalination solution, ensuring efficiency, economic viability, and environmental sustainability.

## Economic viability and energy efficiency of HBD

Energy consumption and total cost are critical determinants of the economic viability and profitability of any technology. These factors are influenced by various elements, including the chosen desalination technology, the type of feed water, the energy source, and the capacity of the desalination plant^334^. To evaluate whether HBD can serve as a viable alternative to conventional water treatment methods, it is essential to compare the processing cost and energy consumption of these methods. When assessing the energy consumption of HBD relative to other desalination techniques, it is crucial to consider the minimum energy required for water desalination as a key benchmark. Regardless of the specific technology or configuration used, theoretical calculations suggest that the minimum energy needed is ~0.7 $\frac{{\rm{kWh}}}{{{\rm{m}}}^{3}}$.^335^, Despite its numerous advantages, HBD suffers from high capital costs, which are greater than those of conventional methods such as reverse osmosis (RO). This underscores the importance of achieving minimal energy consumption. As discussed in the hydrate formers section, utilizing hydrate formers that perform under more favorable conditions can help reduce process energy consumption. For instance, according to He et al.’s investigation^300^, the energy consumption per unit time is 0.05 kW for CP and 0.38 kW for C_3_H_8_ because CP does not require compressors for hydrate formation and can form at atmospheric pressure. Javanmardi et al.^34^ reported an energy expenditure of 25.82 $\frac{{\rm{MJ}}}{{{\rm{m}}}^{3}}$, which is ~7.17 $\frac{{\rm{kWh}}}{{{\rm{m}}}^{3}},$ for the C_3_H_8_ hydrate formation. However, it is generally observed that the total amount of thermal and electrical energy required in the HBD is ~1.58 $\frac{{\rm{kWh}}}{{{\rm{m}}}^{3}}$^336^,

Notably, it is widely reported that LNG offers a substantial and readily available source of cold energy that can significantly reduce both the energy consumption and operational costs of HBD. Integrating LNG with HBD systems offers a promising route to achieving optimal energy efficiency, making it particularly suitable for countries that import large volumes of natural gas in the form of LNG, such as Singapore, China, India, Japan, and South Korea^1,28,29,337^. During LNG production, natural gas is first purified and then liquefied at ~111 K through an energy-intensive process, leading to storage of a considerable amount of cold energy in LNG. The resulting LNG is transported in its liquid state to receiving terminals, where it undergoes regasification, requiring heating to convert liquid (111 K) to gas phase (298 K), typically using seawater as the thermal medium^30,338^. This process releases ~104.5 kWh of cold energy per cubic meter of LNG re-gasified^30^. Under conventional operations, the cooled seawater is discharged back into the ocean, resulting in the loss of a significant amount of recoverable thermal energy. A typical HBD process, as an exothermic process requires external refrigeration cycles, which are energy-intensive and costly, to not only to cool the feed water and hydrate former to a low temperature, but also to remove the heat generated during the hydrate formation, making the energy consumption of the HBD process fairly high. By integrating HBD with LNG regasification, the economic and energy-consumption of the process can be improved. In fact, using heat exchangers to transfer cold energy from LNG into various streams relevant to HBD processes instead of external compressors can significantly reduce the total installed equipment cost of the processes by about 70% and mitigate the operating and maintenance cost of the process^30,34^. In addition, by integrating HBD with LNG regasification, the otherwise wasted cold energy of LNG in its final regasification step can be effectively harnessed to facilitate hydrate formation, thereby eliminating the need for conventional refrigeration cycles^338^. This integration enables HBD to operate at lower temperatures with significantly reduced external energy input and capital expenditure, thus improving both the energy efficiency and economic feasibility of the process^1,30,339^.

Building on this concept, He et al.^1,30^ proposed an innovative method for HBD utilizing LNG cold energy. This approach not only significantly reduced the energy consumption from 65.13 to 0.84 $\frac{{\rm{kWh}}}{{{\rm{m}}}^{3}}$, a 98.71% reduction, with the recovery of hydrate former, but also highlighted its economic viability by reducing the desalination cost from 9.31 to 1.1 $\frac{{\rm{USD}}}{{{\rm{m}}}^{3}}$. Using LNG not only functions as cold energy provider through a refrigerant compression-expansion cycle, but also creates the low-temperature conditions required for the process through a heat exchanger, which makes use of the energetic cold released during the LNG gasification process^316^. Lee et al.,^31^ explored a new process design incorporating an extra expander that utilized the high-pressure gas generated by the dissociation of hydrates to drive the expander, thereby generating electricity. This innovation improved the energy consumption and offered a more cost-effective water treatment solution, leading to a 73% reduction in product cost. Based on their reports, the optimized SEC was ~5.202 $\frac{{\rm{kWh}}}{{{\rm{m}}}^{3}}$ of pure water, and the product cost was 0.148 $\frac{{\rm{USD}}}{{{\rm{m}}}^{3}}$ of pure water. In comparison, conventional methods like RO, which is the state-of-the-art desalination process in the market, has a specific energy consumption value of 4–6 $\frac{{\rm{kWh}}}{{{\rm{m}}}^{3}}$ and a product cost of about 0.7 $\frac{{\rm{USD}}}{{{\rm{m}}}^{3}}$, thus positioning LNG-based HBD as an outstanding alternative desalination approach. Recently, Fernandes et al.,^299^ simulated a seawater HBD process using CO_2_ considering two options for heat/cooling supply. The first option involved a refrigeration cycle/heat pump using ammonia as the cooling fluid, while the second assessed the use of LNG as the cold utility and seawater as the hot utility. In the first case, the total cost of water was estimated at ~3.71 $\frac{{\rm{USD}}}{{{\rm{m}}}^{3}}$, originally reported 3.29 $\frac{{\euro}}{{{\rm{m}}}^{3}}$^299^, with capital expenditures (CapEx) and operating expenses (OpEx) of 1.57 and 2.17 $\frac{{\rm{USD}}}{{{\rm{m}}}^{3}}$, originally reported 1.38 and 1.92 $\frac{{\euro}}{{{\rm{m}}}^{3}}$^299^, respectively, and a specific energy consumption (SEC) of 13.2 $\frac{{\rm{kWh}}}{{{\rm{m}}}^{3}}$. The main costs of the process were associated with the ammonia refrigerant circuits. However, in the second process, the use of LNG as the cold utility led to a total cost of 1.53 $\frac{{\rm{USD}}}{{{\rm{m}}}^{3}}$, originally reported 1.36 $\frac{{\euro}}{{{\rm{m}}}^{3}}$^299^, with CapEx and OpEx of 0.91 and 0.62 $\frac{{\rm{USD}}}{{{\rm{m}}}^{3}}$, originally reported 0.81 and 0.55 $\frac{{\euro}}{{{\rm{m}}}^{3}}$^299^, respectively, and an SEC of 3.8 $\frac{{\rm{kWh}}}{{{\rm{m}}}^{3}}$.

**Fig. 8:**
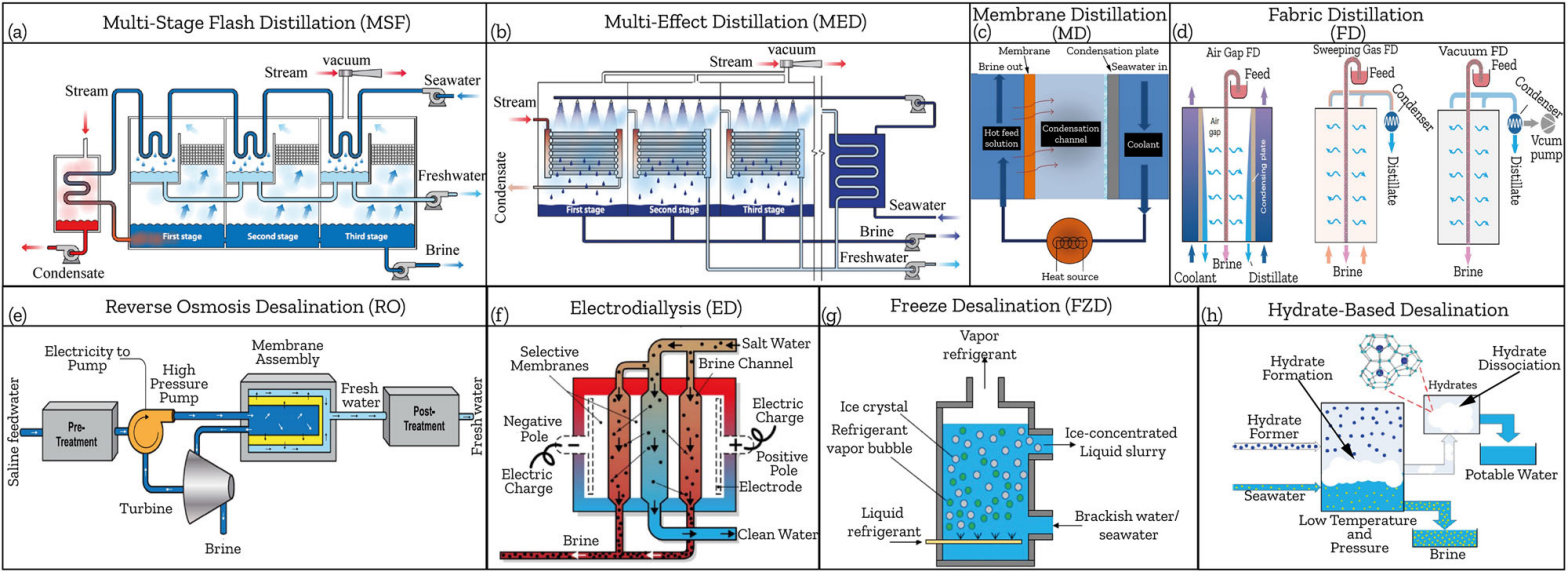
Overview of Desalination Technologies. Schematic representation of various desalination methods, illustrating key desalination technologies, including **a** multistage flash desalination (MSF)^46^, **b** multi-effect distillation (MED)^46^, **c** membrane distillation (MD)^457^, **d** fiber distillation (FD)^400^, **e** freeze desalination (FZD)^427^, **f** electrodialysis desalination (ED)^458^, **g** reverse osmosis (RO)^46^, and **h** hydrate-based desalination (HBD)^51^.

Figure 8 provides schematic representations of advanced and emerging water treatment technologies. Furthermore, Table 5, systematically evaluates critical performance metrics, including energy consumption, water recovery efficiency, salt rejection rates, economic feasibility, and operational parameters, for both conventional and cutting-edge desalination approaches, encompassing evaporative, membrane-based, freeze desalination, and hydrate-based desalination (HBD). Overall, Fig. 8 offers a structured overview of desalination processes, while the accompanying table, Table 5, presents a comprehensive evaluation of key performance metrics—including energy consumption, water recovery efficiency, salt rejection rates, cost analysis, and operational conditions. Together, they provide valuable insights into the strengths and limitations of various desalination technologies.

**Table 5:** Comparative evaluation of HBD and prevalent desalination methods, including an evaluation of key factors such as energy requirements, water recovery yield, salt rejection efficiency, cost, and operating condition ranges

Desalination methods (1*)		Specific energy consumption ($\frac{{\rm{kWh}}}{{{\rm{m}}}^{3}}$)	Water recovery (%)	Salt rejection (%)	Cost (*2) $(\frac{{\rm{USD}}}{{{\rm{m}}}^{3}})$	Operating conditions range		Advantages	Disadvantages	Ref.
						Pressure (bar)	Temperature (K)			
MSF (1*)		13.5–27.25	10–20	>99	0.56– 1.75	(*3)	At top brine temperature 363K-393 K	Reduced scaling issues, High-purity water production, Great efficiency for high, salinity levels, Simplified operation and management	High energy consumption, Low water recovery, High capital and operational costs, Great energy demand, Environmental impact, Elevated operating temperatures.	^30,46,108,290,294,387–393^
MED (1*)		8–21.35 (*2)	20–35	>99	0.52–1.60	(*4)	333–343 K	High efficiency for high TDS solutions, Effective utilization of waste heat, Lower energy consumption compared to MSF, Lower operational temperatures than MSF	Scaling issues, Significant energy use, Low water recovery, Environmental impact, High initial capital cost, Complex maintenance, Need pre-treatment	^31,290,387,388,391,394–399^
MD (1*)		39–67 (*5)	>80 (*6)	>99.5 (For non-volatile solutes)	8.7–30	Vacuum pressure: 0.05–0.35	Feed temperature: 323 K–363 K Cold permeate flow temperature: 278 K–298 K	Utilizes low-grade or waste heat for operation, High salinity rejection, nearly complete removal of salts and non-volatile contaminants, Effective desalination of solution with high TDS (up to or above 200,000 mg/L), Capable of operating in direct contact membrane distillation (DCMD) configuration, which is the simplest and most common setup for desalination, Modular technology, Simple design and operation, Mild operating conditions, Stable performance even at high contaminant concentrations and high salinity levels, Lower environmental impact due to energy efficiency and compatibility with renewable energy sources	High energy consumption, High cost, Wetting and fouling risk, Mineral scaling, less accessible and more expensive membranes compared to FD, Low water recovery, High conductive heat loss, Low water flux, Ineffective heat recovery and temperature polarization limit, Limited large-scale feasibility, Sensitive to feedwater composition	^391,397,400–424^
FD (1*)		Specific thermal energy consumption: 847–24861 (*7)	85	$\approx 100\, \%$ (For non-volatile solutes)	N/A	For VFD: 0.03–0.1	Feed temperature: 313 K-353K Coolant Temperature: 293 K	Effective water transport and vapor permeation due to using hydrophilic fabrics, Utilization of low-grade energy, Small footprint and modular applications, Desalination of a wide range of salinities, Lower cost and use of commonly available, low-cost materials, High water recovery, Higher flux than MD, Simplicity of design and operation	Unable to operate in direct contact configuration, periodic salt removal, Poor mechanical strength and durability, Potential membrane fouling, though less severe than MD, Sensitivity to feedwater composition, especially organic and particulate matter, Ineffective heat recovery, Possible scaling issues, though less severe than MD, Need for optimization in module design to improve performance and lifespan	^400,425,426^
FZD (1*)		11.90–13.78	0–20	Up to 99.7	0.34–0.93	1.03	<270 K	Low chemical use, Low corrosion issues, Reduced scaling and fouling, Environmentally friendly, Versatile feedwater adaptability, Low operating pressure and maintenance costs, High water recovery rate, Low energy requirement, Minimal pretreatment required	High energy demand, Complex ice separation process, Operational complexity, Limited commercial development, Energy recovery limitations, Risk of hydrate formation and contamination, Low water recovery (relative to some methods)	^32,51,336,427–433^
ED		2.64–5.50 (0.7–2.5 at low TDS) (*8)	50-90	Typically, 50–90 (*8)	0.6–1.86	N/A	N/A	Lower energy requirement and capital cost compared to thermal desalination methods, No need for high external hydraulic pressure, unlike RO, Higher water recovery rates, Reduced membrane fouling and scaling compared to RO, Effective for selective removal of ions, Modular and scalable design, Good performance with brackish and moderately saline waters,	Limited Applicability and Cost-Effectiveness, Electrode and Membrane Durability Issues, Selective Removal of Ionic Contaminants and ineffective at removing non-ionic contaminants, Operational Complexity and High Brine Recirculation Requirements	^46,290,391,434,435^
RO (1*)	Conventional RO (1*)	-With recovery tool: 2.1–7.14 -Without recovery tool: 3.66-34.57 (*9)	30–80 (*10)	Up to 99.84	0.36–2.46	10–85	283–318 K	Energy efficient, No thermal energy demand without thermal pollution, No or minimal chemical additives required, Modular and scalable design, Compact system design with a small physical footprint, Technically mature and widely adopted technology, High water recovery rates, Low capital and operating costs, Automated and relatively simple operation, Versatile applications, Effective removal of a broad range of contaminants, Wide availability of membranes and components,	Limited salinity range, High-pressure requirements, High fouling and scaling propensity, Sensitive to feedwater quality and impurities, Limited boron and some contaminant rejection, Membrane damage risk, Environmental impact, Limited applicability for minimum and zero liquid discharge (MLD/ZLD), Requires pretreatment	^31,46,51,388,406,436–442^
	HPRO (1*)	From 125 to 140 bar^439^: 4.41–9.97 (with HER) 7.68–43.21 (without HER)	35-85	Up to 99.69	N/A	Up to 150	283–318 K	concentrate brines up to a maximum of 135 g/l TDS	Membrane compaction and embossing	^439,440,443^
	UHPRO (1*)	From 260–300 bar^439^: 9.18–18.67^439^ (with HER) 16.03–74.07^439^ (without HER)	35–85	Up to 99.47	N/A	Up to 400	283–318 K	Energy efficient Modular Minimal stages required	Membrane compaction and embossing	^439,440^
	LSRO (an N-stage system) (1*)	3–15 (*11)	32–68 (*12)	multi-stages: >99	levelized cost of water (LCOW), TDS 75-150 g/l: 1.74–15.2 (*13)	41.4–85 (Typically <70 bar)	283–318 K (particularly 298 K)	High water recovery, applicable in advancing minimum and zero liquid discharge (MLD/ZLD) systems, optimize pressure requirements and energy efficiency, applicable for a wide range of salinities, particularly for low-salinity feed waters,	membrane fouling and scaling, higher cost than conventional RO	^444–446^
	OARO (an N-stage system) (1*)	5–20	25–85 (*14)	N/A	LCOW: 2.7–6.6 (*14)	For conventional RO: <85 For HPRO: <120	283–318 K	Applicable for a wide range of salinities, particularly for high-salinity feed waters, High Water Recovery Rate, Lower Operating Pressures compared to conventional RO, Energy Efficiency, Potential for Reduced Membrane Fouling, Potential for Brine Management and applicability in advancing MLD/ZLD systems, Capability to Integrate with Renewable Energy,	Membrane Fouling and Scaling, Limited Commercial Availability, Complex System Design, Cost Considerations, Process Optimization Challenges	^444,446–448^
HBD	Conventional HBD	1.4(1.58)–99.96	30–70	60.5–99.8	0.63–4.23	Typically ≥1.03	Typically ≥273.15 K	Low maintenance requirements, Cost-effective, High water recovery rates, Effective treatment of high total dissolved solids (TDS), High corrosion resistance, Energy-efficient operation, Selective separation capability, environmentally friendly process.	High initial capital investment, System complexity, Challenges in hydrate thermodynamics and kinetics, Limited commercial development and scalability, Dependence on hydrate promoters or additives, Environmental concerns related to chemical reliance (like hydrate forms and promoters), Difficulties in recycling integrated chemical components (such as hydrate forms and promoters), Challenges in hydrate-brine separation, Salt deposition on hydrate crystals and difficulty in removal	^1,28,31,34,40,41,336,449,450^
	HBD + (LNG)	0.57–0.84			0.48–1.61					
	HBD + (LNG) + (expanders)	−5.202			0.07–0.547					

(*1) Multistage Flash Desalination (MSF), Multi-Effect Desalination (MED), Membrane Distillation (MD), Fiber Distillation (FD), Freeze Desalination (FZD), Electrodialysis Desalination (ED), Conventional Reverse Osmosis (RO), High-Pressure Reverse Osmosis (HPRO), Ultra-High-Pressure Reverse Osmosis (UHPRO), Low-Salt-Rejection Reverse Osmosis (LSRRO), Osmotically Assisted Reverse Osmosis (OARO), Hydrate-Based Desalination (HBD), and HBD with novel designs.

(*2) Desalination costs depend heavily on both the plant capacity and the process employed. For instance, for MSF seawater desalination, at capacities ranging from 50,000 to 880,000 m^3^/day, the cost is estimated to be between 0.56 and 1.75 USD/ m^3^. Similarly, for MED seawater desalination, plants operating at capacities between 91,000 and 320,000 m^3^/day incur costs of ~0.52 to 1.01 USD/m^3^, those with capacities ranging from 12,000 to 55,000 m^3^/day face costs in the range of 0.95–1.5 USD/m^3^, and plants with capacities below 100 m^3^/day may experience costs as high as 2.0 to 8.0 USD/m^3^^46^.

(*3) MSF desalination systems operate with progressively decreasing pressures across stages to enable the flashing process, for example, 0.7 bar for the first stage and 0.07 bar for the last stage of desalination

(*4) MED systems maintain a pressure gradient across stages, with each stage operating at a lower pressure than the previous one to drive evaporation and condensation. Pressure differences typically range between 5–50 kPa, with less than 5 kPa per stage being common^394^.

(*5) The SEC for direct contact membrane distillation (DCMD) typically falls within the range of 40–45 kWh/m³^421^. In fact, SEC related to MD can be varied from 1 to 9000 kWh/m^3^ which the prominent part of this energy is related to the required thermal energy, such as system configurations, operating conditions (such as feed temperature), and the degree to which heat recovery is implemented. For example, SEC of a membrane distillation unit coupled with a DCMD using solar energy ranges from 200 to 1000 kWh/m^3^, or in another solar-assisted DCMD investigation SEC varies from 380 kWh/m³ in the summer to 2800 kWh/m³ in winter^415,451^.

(*6) DCMD has been reported to achieve 98% water recovery for fractionated effluent and 38% for ozonated effluent^452^, although severe membrane scaling occurs above 80% recovery, while recovery levels up to 70% can be sustained over 24 h without observable scaling^453^.

(*7) STEC for Air Gap FD: 1944–24861 kWh/m^3^, for Sweeping Gas FD: 1292–1872 kWh/m^3^, and for Vacuum FD: 847–1792 kWh/m^3^.

(*8) By extending the ED process duration from 30 to 180 min, the removal efficiency significantly improved from 55.13% to 99.45%. However, this increased treatment time also resulted in a higher specific energy consumption (SEC), rising from 1.02 to 6.10 kWh/m^3^.

(*9) The SEC of RO is primarily influenced by two fundamental operational parameters: operating pressure and process recovery. For example, SEC related to SWRO at 60-80 bar can be varied from 2.1-34.57, the lowest occurs at pressure of 60 bar with the process recovery of 50% with using high recovery energy device leading to SEC equal to 2.1 kWh/m^3^, while 34.57 kWh/m^3^ is SEC of a desalination process occurs at 80 bar with recovery of 10% without using high recovery device^439^.

(*10) HER stands for High Energy Recovery tool.

(*11) At 80 bar, maximum water recovery decreases sharply with rising TDS levels, achieving 89% at 10,000 mg/L, 63% at 35,000 mg/L, and 26% at 70,000 mg/L, highlighting how higher TDS concentrations significantly reduce recovery efficiency^444^.

(*12) 3-stage LSRRO lead to SEC equal to 2.92–15.74, and 4-stage LSRRO lead to SEC equals to 2.39-7.98^444^. Furthermore, an cost-optimal model of LSRRO under 298 K and pressure ranging from 85 bar in the conventional RO stage, and 65 bar in the LSR stages showed over a broad range of feed concentrations (5 and 220 g/L), recovery rates (30%-90%), and number of stages (1–8), shows the SEC of LSRRO can be varied from 1.1 to 171.2 kWh/m^3^ with a LCOW of 0.32 to 41.5 $/m^3^ ^446^. A three-stage LSRRO exhibited SEC values between 2.92 and 15.74 kWh/m^3^, whereas a four-stage configuration yielded an SEC ranging from kWh/m^3^. Additionally, a cost-optimization model at 298 K, with an 85 bar pressure in the conventional RO stage and 65 bar in the LSR stages, demonstrated that, over feed concentrations of 5–220 g/L, recovery rates of 30–90%, and configurations spanning 1–8 stages, SEC can vary from 1.1 to 171.2 kWh/m^3^, with LCOW between 0.32 and 41.5 $/m³. additionally, it is reported that water recovery at feed TDS of 35 g/L feed is 70%, 70 g/L is 55% recovery, and at 125 g/L is 35%. This could achieve to 90%. Under an applied pressure of ΔP = 80 bar, the LSRRO system achieved maximum water recoveries of 93%, 63%, and 24% at feed concentrations of 10 g/L, 35 g/L, and 70 g/L, respectively^444^.

(*13) Water recovery in LSRRO is strongly influenced by factors such as feed water type. For example, a NaCl solution with 21 g/L TDS yields a 68% recovery, brackish water (BW) at 6.2 g/L TDS achieves 64%, seawater (SW) at 9.4 g/L TDS attains 66%, municipal water (MW) with 6.0 g/L TDS delivers 53%, and produced water (PW) with 13.9 g/L TDS results in only 32% recovery^454^.

(*14) The estimated cost of the optimal osmotically assisted reverse osmosis (OARO) configuration for treating feed salinities of 70–125 g/L total dissolved solids (TDS) at water recoveries between 30–70% ranges from 2.7 to 6.6 USD/m^3^. By utilizing low-cost solar electricity and thermal energy for treating solutions with total dissolved solids (TDS) between 30–125 g/L, the LCOW from OARO ranges from 0.70 to 6.28 USD/m^3^.

Although economic and energy-related comparisons are often emphasized in the evaluation of desalination technologies, such assessments can be biased if all critical factors, particularly desalination kinetics, separation efficiency, and environmental impacts, are not considered concurrently. In this work, these aspects have been comprehensively addressed in their respective sections to provide a balanced and multidimensional evaluation of HBD.

## Challenges and prospects

HBD has emerged as a promising desalination technology, particularly in cold regions^32^. This innovative approach effectively addresses several critical challenges associated with conventional desalination methods, such as poor selectivity, high processing and maintenance costs, low efficiency in treating high TDS water, and various environmental concerns^340–342^. A notable advantage of HBD is its high selectivity, which enables the concentration of dissolved salts within a solution. This characteristic can not only be used to streamline traditionally lengthy processes associated with the liquid mining of valuable materials, such as lithium evaporation ponds^33,343^, but also offers a dual benefit: mitigating water scarcity while facilitating the recovery of critical resources^343^. Additionally, this technology presents a remarkable opportunity to address one of the enduring challenges of global warming by effectively capturing greenhouse gases within its molecular cages. Not only does it mitigate the harmful impact of these pollutants but also repurposes them for desalination processes and as potential energy sources for future needs.

Despite these benefits, the industrial application of HBD faces several technical challenges and environmental concerns^17,32,165^. Key issues include the need for high pressures, substantial capital costs, slow hydrate formation kinetics, salt trapping within hydrate cavities, incomplete removal of hydrates from solutions, and difficulties in recovering additives^1,34,44–48^. These challenges complicate the scale-up of HBD and necessitate innovative solutions to enhance both process efficiency and economic viability. To enhance the viability of HBD on an industrial scale, several strategies have been proposed. The utilization of suitable THPs or novel hydrate formers that can operate under more favorable conditions while addressing greenhouse gas emissions such as CO_2_ and CH_4_, can help reduce the high formation pressure requirements in some cases. Additionally, maintaining operations at lower temperatures and pressures, conditions under which corrosion rates are negligible compared to conventional evaporative technologies (e.g., MSF or MED), can reduce overall expenses and enhance corrosion resistance, thereby mitigating operating and maintenance costs and extending system longevity. As confirmed by Javanmardi et al.,^34^ although conventional HBD necessitates high initial capital investments due to the reliance on expensive compressors, the resulting lower energy requirements and the intrinsic durability against corrosion lead to relatively low maintenance costs, thereby enhancing economic viability. Furthermore, the incorporation of cost-effective energy resources such as LNG, as comprehensively discussed in previous sections, is anticipated to further reduce the energy demands and associated expenditures of HBD processes, thereby offsetting the high initial investment and positioning HBD as a promising alternative to conventional technologies^30^.

Controlling the kinetics of hydrate formation and addressing slow formation rates are critical challenges. To meet these objectives, several strategies have been proposed, including the incorporation of KHPs and the development of innovative apparatus designs. Recent advancements aimed at accelerating hydrate formation rates have yielded numerous successful approaches for the sustainable implementation of HBD, with some cases achieving hydrate formation in mere minutes and exhibiting negligible nucleation times. Additionally, the inclusion of KHPs, especially MNBs as an example of these modern solutions, offers practical and cost-effective solutions to address long-standing challenges associated with the implementation of HBD as a scale-up technology in industrial applications^25–27,344^.

The efficient separation of hydrate crystals from unreacted brine and the removal of salts entrapped within hydrate structures remain critical barriers to the further advancement of HBD^35,345,346^. Residual salts trapped within or adsorbed onto hydrate surfaces can degrade hydrate stability and reduce the process’ overall desalination performance. To address these issues and enhance removal efficiency, various separation techniques, such as filtration, washing, centrifugation, and hydrate pelletizing, have been proposed, collectively representing a multifaceted strategy to overcome a pivotal obstacle in scaling up HBD^219,346,347^. For instance, hydrate pelletizing, as demonstrated by Park et al.,^7^ involves producing agglomerated hydrate pellets that facilitate easier handling and improved salt removal compared to conventional techniques. However, salt deposition on pellet surfaces remains a challenge, which hybrid methods combining pelletizing and washing have begun to mitigate. Although this method may be prone to challenges such as salt deposition on hydrate surfaces, hybrid approaches, such as combining pelletizing with washing, have shown promising results in mitigating these drawbacks^347–349^. More recently, Khan et al.^350^ introduced an innovative overflow separation technique using concentric cylinders that effectively prevents salt overflow on hydrate surfaces and within interstitial brine, thereby enhancing separation efficiency^346^. Consequently, these advancements, coupled with the environmentally beneficial effects of HBD, position this method as a compelling and sustainable solution for scale-up water desalination efforts, particularly in regions grappling with acute freshwater scarcity^351–353^.

While challenges remain in the development and implementation of HBD technologies, ongoing research, innovation, and sustained investment are actively working to resolve these issues. With continued progress in both understanding and technological development, HBD is poised to become a cornerstone of sustainable resource management strategies, aligning seamlessly with global environmental objectives. The potential of HBD not only addresses immediate water needs but also contributes to broader climate change mitigation efforts, making it an essential component in the pursuit of a sustainable future.

## Conclusions

Hydrate-based desalination (HBD) has emerged as an innovative, sustainable, and energy-efficient solution to the pressing water scarcity challenges faced by numerous regions worldwide. This review paper summarizes the fundamental properties of hydrates, highlighting their potential as a viable alternative to conventional desalination methods. Despite its promising attributes, HBD encounters significant challenges that impede its commercialization, such as the unfavorable formation conditions associated with some traditional hydrate formers. To address these issues, researchers have proposed various innovative solutions, which this article reviews in depth. We conducted thorough research on recent scientific investigation into diverse hydrate formers and modern, environmentally friendly thermal hydrate promoters (THPs) to optimize hydrate formation conditions. Additionally, a comprehensive analysis of novel kinetic hydrate promoters (KHPs), such as micro-nanobubbles (MNBs) and nanoparticles, aimed at overcoming the slow kinetics that currently limit hydrate formation rates was conducted. This study also evaluates the overall efficiency of HBD in terms of energy consumption and cost-effectiveness. While the initial capital investment for HBD technology may be high, leveraging functional chemical substances for hydrate formation, utilizing cost-effective and practical hydrate formers, and integrating new and inexpensive cold energy sources, such as solar power or liquefied natural gas (LNG), can significantly reduce energy requirements. These strategies can bring energy consumption close to the theoretical minimum for desalination compared to other methods. Ultimately, this reduction in operating costs, combined with low maintenance expenses due to the high corrosion resistance of the process, positions HBD as a viable option for sustainable desalination. In conclusion, HBD is poised to play a transformative role in the future of the desalination industry, offering an innovative approach to addressing global water scarcity challenges while promoting environmental sustainability.

## Data Availability

No datasets were generated or analyzed during the current study.
